# Disrupted circadian oscillations in type 2 diabetes are linked to altered rhythmic mitochondrial metabolism in skeletal muscle

**DOI:** 10.1126/sciadv.abi9654

**Published:** 2021-10-20

**Authors:** Brendan M. Gabriel, Ali Altıntaş, Jonathon A. B. Smith, Laura Sardon-Puig, Xiping Zhang, Astrid L. Basse, Rhianna C. Laker, Hui Gao, Zhengye Liu, Lucile Dollet, Jonas T. Treebak, Antonio Zorzano, Zhiguang Huo, Mikael Rydén, Johanna T. Lanner, Karyn A. Esser, Romain Barrès, Nicolas J. Pillon, Anna Krook, Juleen R. Zierath

**Affiliations:** 1 Department of Physiology and Pharmacology, Integrative Physiology, Karolinska Institutet, Stockholm, Sweden.; 2 Aberdeen Cardiovascular and Diabetes Centre, The Rowett Institute, University of Aberdeen, Aberdeen, UK.; 3 Novo Nordisk Foundation Center for Basic Metabolic Research, Faculty of Health and Medical Sciences, University of Copenhagen, Copenhagen, Denmark.; 4 Department of Molecular Medicine and Surgery, Integrative Physiology, Karolinska Institutet, Stockholm, Sweden.; 5 Department of Physiology and Functional Genomics, College of Medicine, University of Florida, Gainesville, FL, USA.; 6 Department of Biosciences and Nutrition (BioNut), Karolinska Institutet, Stockholm, Sweden.; 7 Department of Physiology and Pharmacology, Molecular Muscle Physiology and Pathophysiology, Karolinska Institutet, Stockholm, Sweden.; 8 Institute for Research in Biomedicine (IRB Barcelona), Barcelona Institute of Science and Technology, Barcelona, Spain.; 9 Departament de Bioquímica y Biomedicina Molecular, Facultat de Biologia, Barcelona, Spain.; 10 CIBER de Diabetes y Enfermedades Metabólicas Asociadas (CIBERDEM), Instituto de Salud Carlos III, Spain.; 11 Department of Biostatistics, University of Florida, Gainesville, FL, USA.; 12 Department of Medicine (H7), Unit for Endocrinology and Diabetes, Karolinska Institutet, Stockholm, Sweden.

## Abstract

Circadian rhythms are generated by an autoregulatory feedback loop of transcriptional activators and repressors. Circadian rhythm disruption contributes to type 2 diabetes (T2D) pathogenesis. We elucidated whether altered circadian rhythmicity of clock genes is associated with metabolic dysfunction in T2D. Transcriptional cycling of core-clock genes *BMAL1, CLOCK*, and *PER3* was altered in skeletal muscle from individuals with T2D, and this was coupled with reduced number and amplitude of cycling genes and disturbed circadian oxygen consumption. Inner mitochondria–associated genes were enriched for rhythmic peaks in normal glucose tolerance, but not T2D, and positively correlated with insulin sensitivity. Chromatin immunoprecipitation sequencing identified CLOCK and BMAL1 binding to inner-mitochondrial genes associated with insulin sensitivity, implicating regulation by the core clock. Inner-mitochondria disruption altered core-clock gene expression and free-radical production, phenomena that were restored by resveratrol treatment. We identify bidirectional communication between mitochondrial function and rhythmic gene expression, processes that are disturbed in diabetes.

## Teaser

Disturbances in daily rhythms of mitochondrial activity may contribute to skeletal muscle insulin resistance in type 2 diabetes.

## INTRODUCTION

Type 2 diabetes (T2D) is a growing global health problem, with skeletal muscle insulin resistance being a primary defect in the pathology of this disease. While the etiology of this disease is complex, perturbed sleep/wake rhythms from shift-work, sleep disorders, and social jet lag are associated with obesity, T2D, and related comorbidities (*1*–*4*), highlighting the critical role of the circadian timing system for metabolic health. Cell autonomous circadian rhythms are generated by a transcription-translation autoregulatory feedback loop composed of transcriptional activators CLOCK and BMAL1 (*ARNTL*) and their target genes Period (*PER*), Cryptochrome (*CRY*), and REV-ERBα (*NR1D1*), which rhythmically accumulate and form a repressor complex that interacts with CLOCK and BMAL1 to inhibit transcription (*5*). Disruption of the molecular clock in skeletal muscle leads to obesity and insulin resistance in mouse models (*6*–*8*). While disrupted circadian rhythms alter metabolism, the extent to which these processes are impaired in people with T2D is unknown.

Several lines of evidence suggest that the link between dysregulated molecular-clock activity and T2D or insulin resistance may be tissue dependent. In white adipose tissue, the evidence is equivocal. For example, subcutaneous white adipose tissue biopsies showed no difference of rhythm and amplitude of core-clock (*PER1*, *PER2*, *PER3*, *CRY2*, *BMAL1*, and *DBP*), clock-related (*REVERB*α), and metabolic (*PGC1*α) genes between individuals with normal weight, obesity, or T2D over a time-course experiment (*9*). Conversely, when the sleep/wake cycle and dietary regime are controlled, amplitude oscillations of core-clock genes and number of rhythmic genes are reduced in adipose tissue from people with T2D as compared with healthy, lean individuals (*10*). In human leukocytes collected over a time-course experiment, mRNA expression of *BMAL1*, *PER1*, *PER2*, and *PER3* was lower in people with T2D as compared to nondiabetic individuals (*11*). In addition, *BMAL1*, *PER1*, and *PER3* mRNA expression in leukocytes collected from people with T2D is inversely correlated with hemoglobin A_1C_ (HbA_1c_) levels, suggesting an association of molecular-clock gene expression with T2D and insulin resistance. Furthermore, in pancreatic islets from individuals with T2D or healthy controls, *PER2*, *PER3*, and *CRY2* mRNA expression is positively correlated with islet insulin content and plasma HbA_1c_ levels (*12*). Thus, there may be tissue specificity of molecular-clock regulation, which contributes to clinical outcomes related to insulin sensitivity and T2D etiology. The underlying mechanisms regulating metabolic rhythmicity and, particularly, whether rhythmicity is lost in T2D remain incompletely understood.

At the cellular level, primary human myotubes maintain a circadian rhythm, with the amplitude of the circadian gene *REV-ERB*α correlating with the metabolic disease state of the donor groups (*13*). This apparent link between the skeletal muscle molecular clock and insulin sensitivity may be partly mediated by molecular-clock regulation of metabolic targets. Chromatin immunoprecipitation (ChIP) sequencing has revealed distinct skeletal muscle–specific BMAL1 and REV-ERBα cistromes (*14*), with prominent molecular clock–targeted pathways, including mitochondrial function and glucose/lipid/protein metabolism (*14*, *15*). Moreover, these metabolic pathways may participate in retrograde signaling to control aspects of the molecular clock. Pharmacological inhibition of *DRP1*, a key regulator of mitochondrial fission and metabolism, alters the period length of BMAL1 transcriptional activity in human fibroblasts (*16*). However, the signals and the clock-derived alterations that govern the rhythmicity of metabolism remain incompletely understood. Despite the growing evidence that several metabolic pathways are under circadian control, it is not clear whether circadian rhythmicity of the intrinsic molecular clock is altered in T2D. Here, we determined whether circadian control of gene expression and metabolism is altered at the cellular level in skeletal muscle from individuals with T2D.

## RESULTS

### Intrinsically dysregulated circadian rhythm of gene expression in T2D

**Fig. 1.**
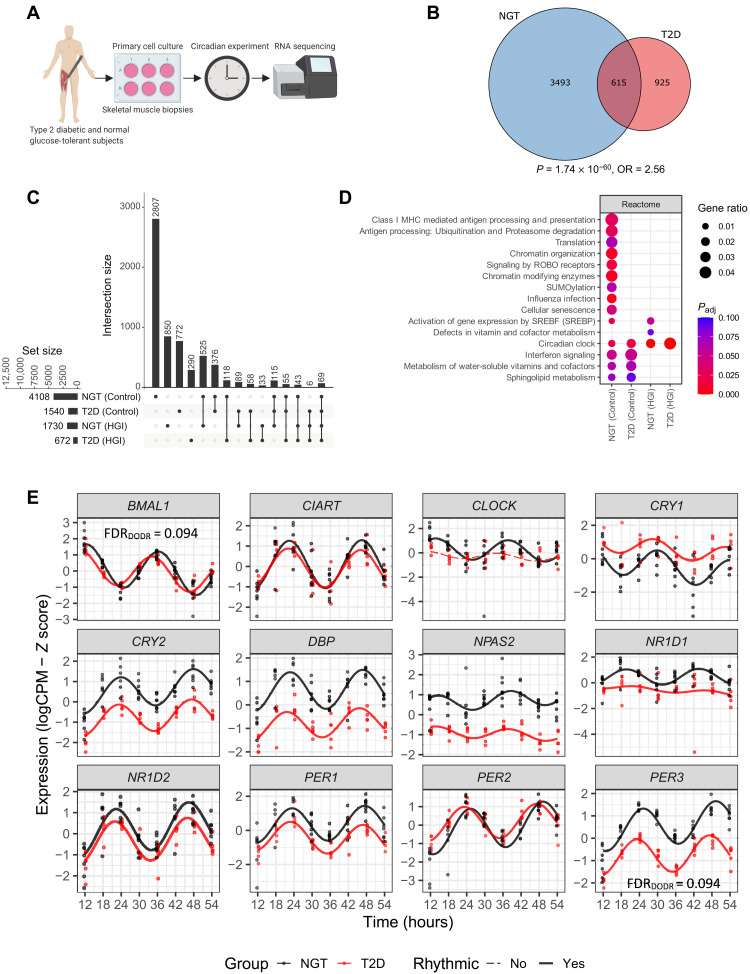
Intrinsically dysregulated circadian rhythm of gene expression in T2D. (**A**) Schematic overview of primary cell culture circadian experiment and RNA sequencing. RNA sequencing of primary human skeletal muscle cells from men with NGT (*n* = 7) or T2D (*n* = 5). (**B**) Venn diagram showing the overlapping rhythmic genes between NGT and T2D. Overlapping rhythmic genes were significantly enriched (Fisher’s exact test, *P* = 1.74 × 10^−60^; odds ratio, OR = 2.56, background = 18,482). (**C**) Upset plot showing number of circadian genes identified via RAIN analysis for each disease and treatment comparison (FDR < 0.10). (**D**) Circadian gene enrichment results using ORA (Fisher’s exact test) and Reactome pathways. Circadian genes identified via RAIN analysis (FDR < 0.10), and the top 10 enriched Reactome pathways are shown (FDR < 0.10). (**E**) Circadian rhythmicity of core clock genes. Red = T2D, black = NGT. Lines show the harmonic regression fits and solid line indicates circadian (FDR < 0.10) genes, while dashed lines indicate noncircadian genes. Time points are hours after synchronization.

Skeletal muscle biopsies were obtained from men with either T2D or normal glucose tolerance (NGT) (Fig. 1A). Primary myotube cultures were prepared, synchronized, and harvested every 6 hours over 42 hours, and the transcriptome was analyzed by RNA sequencing. To determine whether genes displayed a rhythmic cycle over 24 hours, we analyzed the RNA-sequencing expression values using “rhythmicity analysis incorporating non-parametric methods” (RAIN) algorithm with longitudinal method (table S1) (*17*). Myotubes from T2D donors displayed fewer cycling rhythmic genes as compared to myotubes from NGT donors. The rhythmic genes in NGT were also significantly associated with the rhythmic genes in T2D as the overlap between NGT and T2D circadian genes was significant with an odds ratio (OR) greater than 1 (Fig. 1B, Fisher’s exact test, OR = 2.56, *P* = 1.74 ×10^−60^). To induce acute insulin resistance and partly mimic a T2D milieu (*18*), myotubes from NGT and T2D donors were treated with high glucose and insulin (50 nM insulin and 25 mM glucose) for 24 hours before serum shock. Treatment with a high concentration of glucose and insulin reduced the number of cycling genes in myotubes from both T2D (−868 genes, 56.4% reduction) and NGT (−2378 genes, 57.9% reduction) donors (Fig. 1C). We performed Gene Set Enrichment Analysis (GSEA) and found that the Reactome pathway (Fig. 1D) (*19*) “Circadian Clock” was enriched in all conditions. To assess the altered rhythmicity (the so-called differential rhythmicity) between NGT and T2D, the RNA-sequencing expression values from the NGT and T2D donors were compared to each other using an algorithm for detection of differential rhythmicity (DODR) (*17*). *PER3* and *BMAL1* (aka *ARNTL*) were differentially rhythmic between NGT and T2D donors (in total, six genes were shown to be differentially rhythmic between NGT and T2D donors; table S2) [Fig. 1E; both genes false discovery rate (FDR)_DODR_ = 0.094]. The molecular-clock gene *CLOCK* was exclusively cycling in NGT donors (FDR_RAIN_ < 0.1) (Fig. 1E). These results indicate that myotubes from the NGT donors had more genes displaying rhythmic behavior. In addition, two (*BMAL1* and *PER3*) core-clock genes displayed differential rhythmicity (DODR) and one (*CLOCK*) was exclusively rhythmic (RAIN) in myotubes from NGT and T2D donors.

### Reduced amplitudes of rhythmic gene expression in T2D

**Fig. 2.**
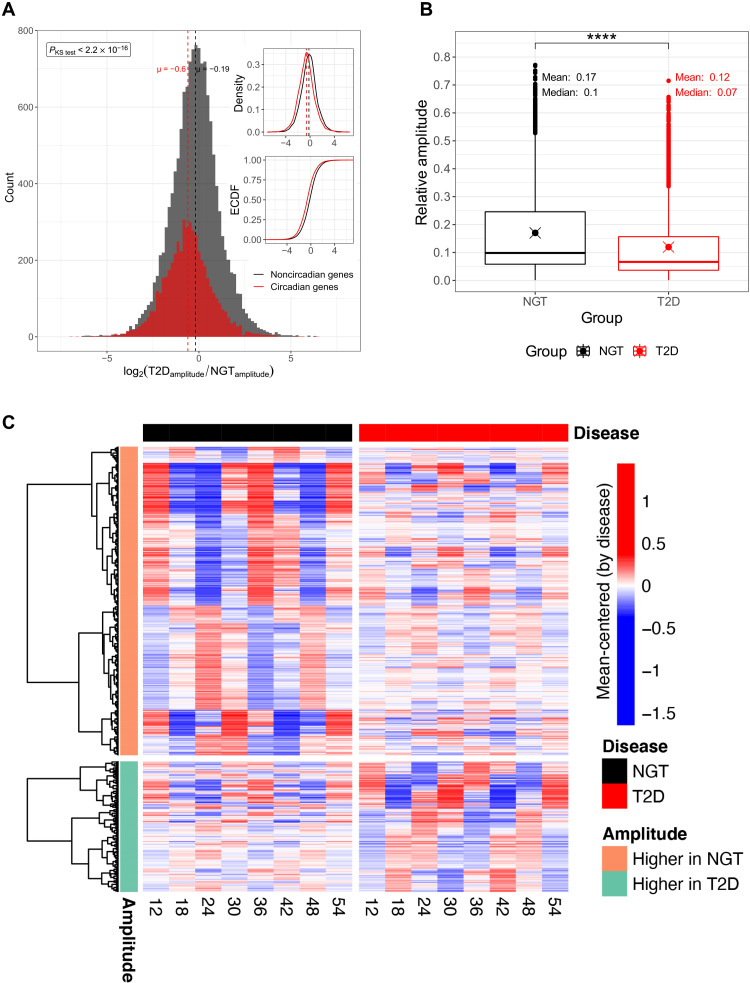
Reduced amplitudes of overall rhythmic gene expression in T2D. (**A**) Log_2_ relative amplitude of circadian genes for T2D as compared to NGT; relative amplitudes were determined via harmonic regression with first-degree polynomial trend using mean-centered data. Histogram of circadian genes (red) and all genes (black), log_2_ relative amplitude of T2D versus NGT. The main panel shows count histogram of log_2_ relative amplitudes of circadian genes (red) and noncircadian genes (black). Right panels show the density distributions and empirical cumulative distribution function. Red = circadian, determined via RAIN algorithm (FDR < 0.1), black = noncircadian. Log_2_ relative amplitude distributions were significantly different in circadian genes compared to noncircadian genes (*P*_KS_ < 2.2 × 10^−16^, two-sided Kolmogorov-Smirnov test). μ = population mean. (**B**) Boxplots showing the relative amplitude comparison between NGT and T2D groups for the union of circadian genes. Means and medians are reported for each group in the plot. Black: NGT, red: T2D groups. The difference between the relative amplitudes are calculated using Wilcoxon rank sum test (****P < 2.2 × 10^−16^). (**C**) Heatmaps showing the amplitude differences of RNA-sequencing gene expression data of NGT and T2D groups. The linear trends are removed for visual purposes to highlight the patterns in data (see Materials and Methods). To show the amplitude differences between NGT and T2D, the data were mean-centered for the groups. Each time point shown in the heatmap is the average value after mean-centering. Hierarchical clustering was performed by using geodesic distance and “ward.D2” algorithm separately for higher amplitude clusters in NGT and T2D groups.

An important factor that is often physiologically relevant in circadian and diurnal biology is the magnitude of cycling peaks and nadirs over the course of a cycle, which can be quantified by measuring the amplitude of cycling patterns. We used harmonic regression to determine relative amplitude of cycling gene expression in myotubes from NGT and T2D donors within the RNA-sequencing dataset (table S3). The log_2_ relative amplitudes of the circadian genes (T2D compared to NGT) were significantly different from noncircadian genes (Fig. 2A; *P* < 2.2 × 10^−16^, two-sided Kolmogorov-Smirnov test), and the mean relative amplitude of cycling genes was lower in T2D as compared to NGT (Fig. 2B; *P* < 2.2 × 10^−16^, paired two-sided Wilcoxon rank sum test). Furthermore, we created heatmaps to better visualize the differences in amplitudes between NGT and T2D groups (Fig. 2C). These heatmaps demonstrated that although most cycling genes had reduced cycling amplitudes in T2D, there were also several that displayed higher amplitudes in T2D compared to NGT.

### Altered peak-time signature of cycling inner-mitochondrial genes and ablated rhythmic mitochondrial metabolism in myotubes from T2D donors

**Fig. 3.**
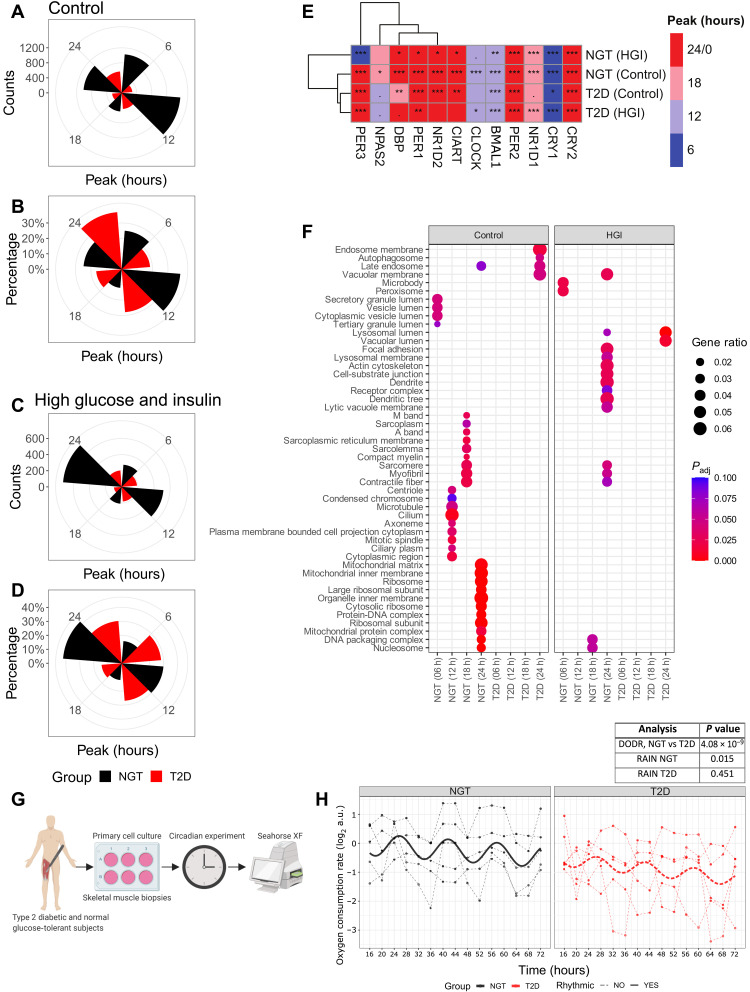
Altered peak-time signature of cycling inner-mitochondrial genes and ablated rhythmic mitochondrial metabolism in myotubes from T2D donors. (**A**) Number of circadian genes at each peak time for control treatments. (**B**) Percentage of circadian genes at each peak time for control treatments. (**C**) Number of circadian genes at each peak time when treated with high concentrations of glucose and insulin. (**D**) Percentage of circadian genes at each peak time when treated with high concentrations of glucose and insulin. (**E**) Heatmap showing the peak times of core clock genes for control conditions and cells treated with high concentrations of glucose and insulin. Colors represent the peak times. Hierarchical clustering was performed by using geodesic distance and “ward.D2” algorithm. Asterisks represent the adjusted *P* values from rhythmicity analysis (RAIN): ***0 ≤ *P* ≤ 0.001; **0.001 < *P* ≤ 0.01; *0.01 < *P* ≤ 0.05; · 0.05 < *P* ≤ 0.1; empty boxes, 0.1 < *P* ≤ 1. (**F**) GO:CC enriched at each time point in NGT (control), T2D (control), NGT (high concentration of glucose and insulin), and T2D (high concentration of glucose and insulin). (**G**) Schematic of circadian basal cellular oxygen consumption rate (OCR) time-course experiment. (**H**) Relative OCR of synchronized myotube cultures from donors with NGT (black) versus T2D (red), as measured by Seahorse XF Analyzer (Agilent) for *n* = 5 donors in each group. Differential rhythmicity (DODR, period = 16 hours) and rhythmicity (RAIN) analysis statistics are shown in table inset. See also fig. S4. Lines show the harmonic regression fits, and solid line indicates rhythmic (FDR_RAIN_ < 0.1) OCR, while dashed lines indicate nonrhythmic OCR (RAIN analysis). The harmonic regression line in the figure is for illustration purposes only and was not used in the statistical analysis. a.u., arbitrary units.

Analyzing the same RNA-sequencing data as described in Fig. 1, we used the RAIN algorithm to determine the peak time of the cycling genes in the myotube cultures. T2D displayed an altered pattern of cycling gene peaks as compared to NGT (Fig. 3, A and B). In percentage terms, T2D had the highest number of cycling genes displaying peaks at 24 hours, whereas NGT had the highest number of cycling genes displaying peaks at 12 hours (Fig. 3B). When myotubes were treated with a high concentration of glucose and insulin (Fig. 3, C and D), the peak at which T2D had the largest amount of cycling genes remained consistent (24 hours), while the highest peak genes in NGT was shifted to 24 hours. When considering the molecular-clock genes and clock-output genes (Fig. 3E), *NPAS2* displayed different peak times between NGT [Zeitgeber time, 18 hours (ZT18)] and T2D (ZT12).

We then performed a gene enrichment analysis [over-representation analysis (ORA)] for each condition and gene peak time. Several mitochondrial Gene Ontology (GO) cellular components (CC) (“mitochondrial matrix,” “mitochondrial inner membrane,” and “mitochondrial protein complex”) displayed enrichment for cycling genes at peak time 24 hours (ZT24) in NGT but not in any other condition (Fig. 3F). Genes displaying peak times at ZT24 in cells from NGT donors were also associated with the Reactome pathway (histone acetyltransferase) “HATs acetylate histones” (fig. S3).

Given that an absence of mitochondrial GO:CC at ZT24 was observed in myotubes from the T2D donors (Fig. 3F) and that T2D is associated with reduced skeletal muscle mitochondrial function and metabolic inflexibility (*20*), we investigated whether synchronized myotubes from NGT and T2D donors displayed circadian oscillations of oxygen consumption rate (OCR, a proxy for mitochondrial metabolism; Fig. 3G). We observed differential rhythmicity of OCR between NGT and T2D (DODR, FDR = 4.08 × 10^−9^) (Fig. 3H). NGT displayed cycling OCR with a 16-hour period (FDR_RAIN_ = 0.015), whereas T2D did not (or any other period). In addition, we performed a separate Mito Stress Test at a single time point (ZT24) after serum shock (fig. S4). In this assay, mitochondrial function did not differ between the myotubes from NGT and T2D donors. Thus, the difference of rhythmic mitochondrial function between the myotubes from the NGT and T2D donors may be derived from temporal regulation of oxygen consumption rather than differences in mitochondrial function per se. Overall, our data (Fig. 3) demonstrate that myotubes from T2D donors have reduced mitochondrial metabolic rhythm of both OCR and mitochondrial-related gene peaks at ZT24.

### Mitochondrial inner membrane is enriched for genes that correlate with whole-body insulin sensitivity

**Fig. 4.**
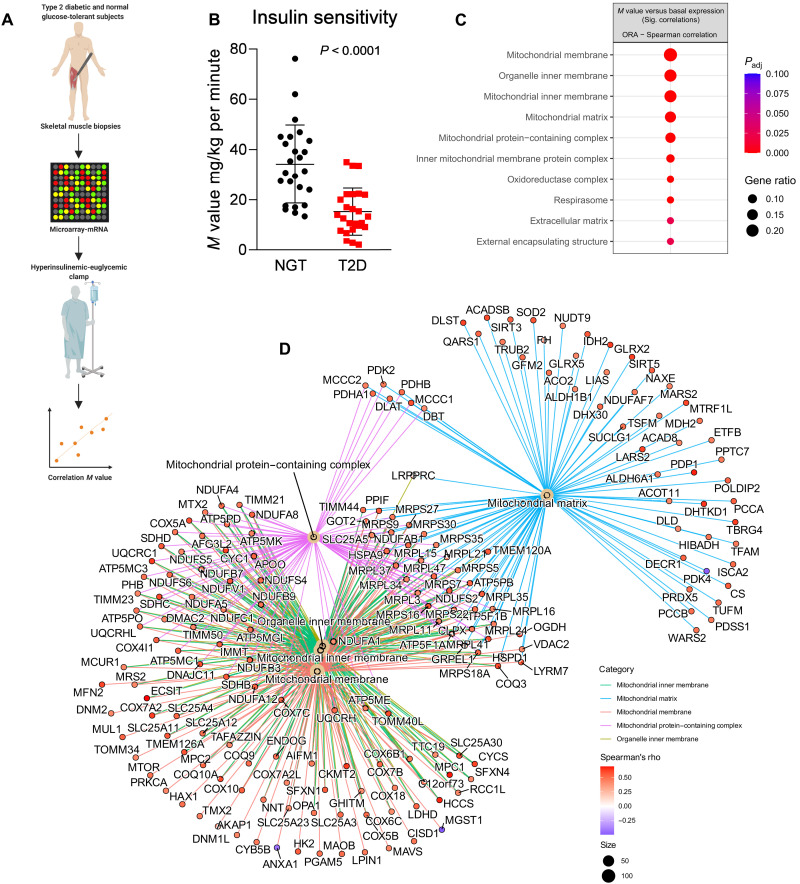
Mitochondrial inner membrane is enriched for genes that correlate with whole-body insulin sensitivity. (**A**) Schematic of experimental design. (**B**) Insulin sensitivity (*M* value: mg glucose infused/kg per minute) of T2D and NGT participants. Student’s *t* test. (**C**) Enrichment analysis based on microarray of skeletal muscle biopsies obtained in the fasted state from 24 men with NGT and 25 men with T2D. Top 10 differentially enriched GO:CC, ranked by gene ratios (FDR < 0.10). Pathway enrichment calculated from genes correlating (FDR < 0.10) with *M* value (whole-body insulin sensitivity) in vivo. (**D**) GO term–gene interaction plot of the top five GO:CC enrichments calculated from genes correlating (FDR *<* 0.1) *M* value (whole-body insulin sensitivity) in vivo: organelle inner membrane, mitochondrial inner membrane, mitochondrial protein complex, mitochondrial matrix, and oxidoreductase complex. Node colors for each gene represent the Spearman correlation coefficient (ρ).

To test the in vivo clinical relevance of our findings in the primary myotube cultures, we performed a transcriptomic analysis on vastus lateralis muscle biopsies obtained from men with NGT (*n* = 24) or T2D (*n* = 25) at a single time point (Fig. 4A). In addition, a hyperinsulinemic-euglycemic clamp was performed to determine whole-body insulin sensitivity (*M* value, analysis pipeline shown in Fig. 4A). As expected, insulin sensitivity was greater in men with NGT as compared to those with T2D (Fig. 4B, *P* < 0.0001). The relationship between basal skeletal muscle gene expression and insulin sensitivity across the whole cohort was assessed by Spearman’s rank correlation (Fig. 4A). A gene enrichment analysis (ORA) with ontology terms from GO:CC was performed using genes significantly correlating (Spearman’s rank correlation) with basal gene expression. We found that most of the enriched GO:CC were related to the mitochondria (FDR < 0.10; Fig. 4, C and D). The GO:CC “mitochondrial matrix” and “mitochondrial inner membrane” were enriched at ZT24 in myotubes from NGT donors (Figs. 3F and 4D). Our analysis points at the inner mitochondria as a cellular location implicated in the regulation of skeletal muscle insulin sensitivity in vivo, as well as the impaired cycling behaviors in skeletal myotubes from T2D donors.

### Mitochondrial pathways are enriched for genes with CLOCK and BMAL1 binding and are associated with whole-body insulin sensitivity

**Fig. 5.**
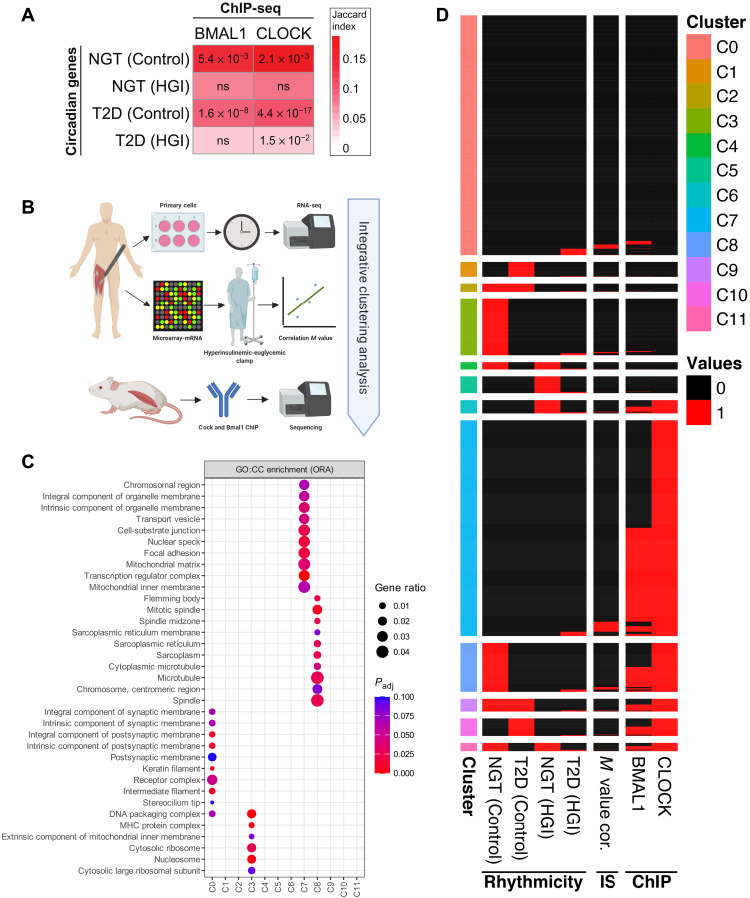
Mitochondrial pathways are enriched for genes with Clock and Bmal1 binding and are associated with whole-body insulin sensitivity. (**A**) Enrichment analysis between circadian genes and ChIP-sequencing peaks. The association between circadian genes in each disease-treatment group and BMAL1/CLOCK bound genes were tested using Fisher’s exact test, and *P* values are adjusted for multiple testing with the Benjamini-Hochberg method. Columns: BMAL1/CLOCK ChIP-sequencing experiments; rows: rhythmic genes in NGT and T2D for both control conditions and high concentration of glucose and insulin treatment groups; numbers: adjusted *P* values (ns: not significant); colors: Jaccard similarity index indicating the percentage of genes overlapping in each dataset. (**B**) Schematic of experimental design used for the integrative analysis. (**C**) Gene enrichment analysis using ORA (Fisher’s exact test) with the top 10 GO:CC was performed (FDR < 0.10) for each cluster identified in (B). (**D**) Heatmap showing the clusters from the integrative analysis. Binary data used for clustering with “clust” algorithm (*67*) and resulted in 12 clusters. Columns “NGT (Control),” “T2D (Control),” “NGT (high concentration of glucose and insulin),” and “T2D (high concentration of glucose and insulin)” represent circadian (black) and noncircadian (white) genes; “*M* value cor” shows whether a significant (black) correlation between the insulin sensitivity metric (*M* value) and basal gene expression; “BMAL1” and “CLOCK” show whether a ChIP-sequencing peak for the given protein was detected (black) or not (white).

In the current study, we describe intrinsically dysregulated rhythm, peak time, and amplitudes of cycling genes in myotubes from T2D as compared to NGT donors. We also observed impaired cycling behavior of mitochondrial-related genes in T2D, coupled with functionally dysregulated mitochondrial rhythmic metabolism. However, the regulatory directionality of these phenomena (i.e., rhythmic dysfunction of the molecular-clock and mitochondrial metabolism) is unclear. Whether both BMAL1 and CLOCK partially regulate mitochondrial metabolism in skeletal muscle is also unclear. We found that these molecular-clock genes were rhythmically dysregulated in myotubes from T2D as compared to NGT donors. Therefore, we tested whether BMAL1 and CLOCK binding was associated with mitochondrial genes involved in insulin sensitivity and dysregulated mitochondrial metabolic rhythms, which would indicate that dysregulated BMAL1 and CLOCK lead to disrupted mitochondrial rhythms in skeletal muscle of people with T2D. We performed ChIP sequencing of mouse skeletal muscle using BMAL1 and CLOCK antibodies and assessed peaks closest to a gene’s transcription start site (TSS) with the highest peak score. First, we assessed whether circadian genes identified in myotubes from NGT and T2D donors (identified by RAIN) were associated with BMAL1 and CLOCK binding to homologous mouse genes (Fig. 5A; Fisher’s exact test, FDR < 0.05; Jaccard index indicates the percentage overlap between circadian genes and BMAL1/CLOCK bound genes). Rhythmic genes of NGT and T2D myotubes were associated with BMAL1 and CLOCK binding, while rhythmic genes of T2D myotubes treated with a high concentration of glucose and insulin were associated with CLOCK binding exclusively. We then performed integrative clustering analysis between the ChIP-sequencing data, skeletal muscle gene expression correlated with insulin sensitivity, and circadian genes identified within myotubes from NGT and T2D donors incubated in the absence or presence of a high concentration of glucose and insulin (Fig. 5B). To integrate all different experimental outcomes, the data were presented as a binary matrix, where 1 shows the presence and 0 shows the absence of rhythmicity, correlation with insulin sensitivity, or ChIP-sequencing peaks. The integrated data were subjected to clustering analysis, which resulted in 12 distinct clusters (Fig. 5D). Clusters associated with BMAL1 and CLOCK binding were also associated with circadian rhythm in NGT (Control: clusters 8, 9, and 11) and T2D [Control: clusters 9 and 10; high glucose/insulin (HGI): a few genes in clusters 6 to 11].

Genes that correlated with insulin sensitivity formed a cluster with BMAL1 and CLOCK binding and circadian genes within myotubes from NGT donors (cluster 8). We then performed GSEA of GO:CC (Fig. 5C). Of note, clusters 3 and 7 were enriched for genes associated with the inner mitochondria. Cluster 7 contained no genes with circadian rhythmicity in NGT or T2D. Cluster 3 was enriched for several cellular compartments, including “extrinsic component of mitochondrial inner membrane,” and had very few genes with BMAL1/CLOCK binding despite all genes in this cluster showing circadian rhythmicity in NGT (but lost in T2D). Thus, mitochondrial rhythmic metabolism within NGT may be partly independent of genes with CLOCK or BMAL1 binding.

### Pharmacological, genetic, and siRNA-mediated disruption of inner-mitochondrial metabolism results in altered clock-gene expression

**Fig. 6.**
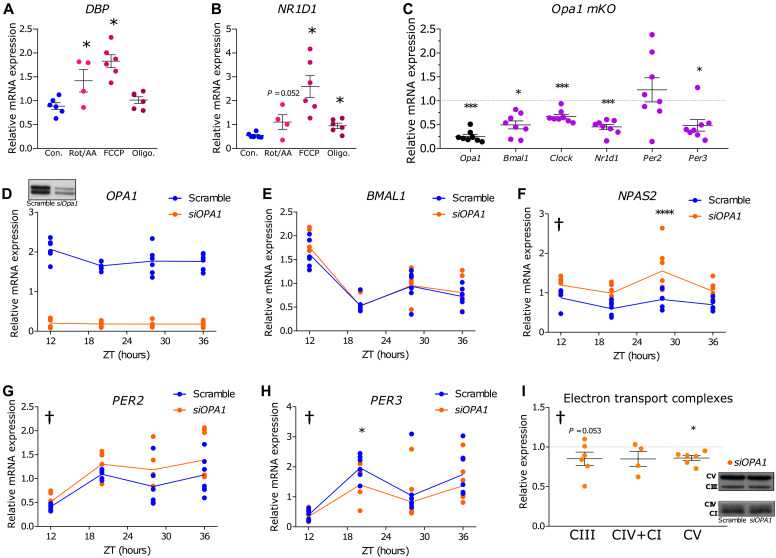
Pharmacological, genetic, and siRNA-mediated disruption of inner-mitochondrial metabolism results in altered clock-gene expression. (**A**) mRNA expression of molecular clock–associated gene DBP in myotubes from NGT donors (*n* = 4–6 donors). One-way analysis of variance (ANOVA), *P < 0.05 compared to vehicle control (Con.). (**B**) mRNA expression of molecular clock–associated gene NR1D1 in myotubes from NGT donors (*n* = 4 to 6 donors). One-way ANOVA, *P < 0.05 compared to vehicle control (Con.). (**C**) *OPA1* and Clock gene expression (*Bmal1, Clock, Nr1d1, Per2,* and *Per3*) from skeletal muscle-specific *Opa1* knockout (*Opa1 mKO*) mice (*n* = 8) relative to control (*n* = 7). Black = *Opa1 mKO* *Opa1* gene expression, purple = core-clock–associated gene expression one-way ANOVA, ***P < 0.001, *P < 0.05. (**D**) *OPA1 mRNA* expression and protein abundance (inset) over the time-course experiment after synchronization in primary human skeletal muscle cells treated with *siRNA* targeting *OPA1* (*siOPA1*) (*n* = 6) compared to Scramble *siRNA* (*n* = 6). (**E** to **H**) Primary human skeletal muscle cells treated with *siOPA1* (*n* = 6). Molecular-clock genes *BMAL1* (E)*, NPAS2* (F), *PER2* (G), and *PER3* (H). †*P* < 0.05 overall difference *siOPA1* versus Scramble. ****P < 0.0001, *P < 0.05 *siOPA1* versus Scramble at time point (two-way ANOVA). Genes are shown in synchronized cells and presented at each ZT after serum shock. (**I**) Protein abundance of electron transport complexes. Orange, *siOPA1* (*n* = 4–6 donors). One-way ANOVA, † = overall effect of *siOPA1* (*P* < 0.05), *P < 0.05 effect of *siOPA1*.

Our data provide evidence that mitochondrial metabolic rhythms in skeletal muscle from individuals with NGT may be partly independent of direct core-clock control. In addition, inner-mitochondrial gene expression was associated with whole-body insulin sensitivity. Targets involved in inner-mitochondrial functionality can act as regulators of molecular-clock expression and function (*16*, *21*). Thus, we hypothesized that internal mitochondrial functionality may play a role in regulating metabolic and molecular-clock rhythms. To test this in myotubes from NGT donors, we used different compounds that target mitochondria, namely, carbonyl cyanide 4-(trifluoromethoxy)phenylhydrazone (FCCP), oligomycin, and a mixture of rotenone and antimycin A (Rot/AA). After serum shock, we incubated myotubes with these compounds for 4 hours (ZT14 to ZT18). We measured mRNA expression of molecular clock–associated genes *DBP* (Fig. 6A) and *NR1D1* (Fig. 6B) and found that 0.33 μM Rot/AA and 2 μM FCCP increased mRNA expression of *DBP* as compared to vehicle control–treated myotubes. In addition, 2 μM FCCP and 1 μM oligomycin increased mRNA expression of *NR1D1* as compared to vehicle control–treated myotubes. These data provide evidence that manipulation of inner-mitochondrial function may play a role in altering clock-associated gene expression in primary human myotubes. However, compounds such as these may have off-target effects, and thus, we tested this hypothesis in other models.

We sought to manipulate a target that both regulated inner-mitochondrial function in myotubes and displayed diurnal behavior in vivo in skeletal muscle biopsies from individuals with NGT. Meeting these criteria, optic atrophy protein 1 (OPA1) is an established regulator of inner-mitochondrial morphology and function in skeletal muscle (*22*–*25*) and displays a day-night change over time of protein abundance in skeletal muscle from healthy individuals alongside oscillating mitochondrial function (*26*). *OPA1* did not display cycling mRNA in myotubes from NGT or T2D donors when adjusted for multiple comparisons. However, *OMA1* and *HIGD2A* are responsible for the cleavage and stabilization of OPA1 protein (*27*, *28*), respectively, and both genes displayed cycling mRNA patterns in myotube cultures from NGT, but not T2D donors (as determined via RAIN; fig. S5 and table S1), suggesting circadian regulation of OPA1 protein. *OPA1* basal gene expression also correlated with insulin sensitivity in the microarray data (Spearman’s correlation, FDR = 0.002; fig. S6). To further elucidate the effect of mitochondrial disruption in relation to the molecular-clock machinery, we performed investigations in a skeletal muscle–specific *Opa1^−/−^* mouse model. Molecular-clock genes *Clock, Bmal1, Nr1d1*, and *Per3* (Fig. 6C) were decreased in *Opa1^−/−^* mice, suggesting retrograde signaling from OPA1 to the molecular clock.

However, the *Opa1^−/−^* mice we studied have a severe myopathic phenotype (*23*), and we thus aimed to recapitulate this finding in myotube cultures from NGT donors (Fig. 6, D to H). In synchronized primary human skeletal myotubes, small interfering RNA (siRNA)–targeted reduction of *OPA1* (Fig. 6D) resulted in unchanged *BMAL1* (Fig. 6E) expression, while *NPAS2* (a paralog and substitute of *Clock*) (*29*) (Fig. 6F), *PER2* (Fig. 6G), and *PER3* (Fig. 6H) all had altered mRNA expression in the OPA1-depleted condition. *OPA1* silencing also perturbed expression of Complex V of the electron-transport chain (Fig. 6I). Collectively, these data suggest that manipulating inner-mitochondrial metabolism results in altered mRNA expression of clock genes.

### Transcription factor–gene network analysis reveals divergent circadian transcription factor enrichment of NPAS2, NFKB1, and RELA between T2D and NGT

**Fig. 7.**
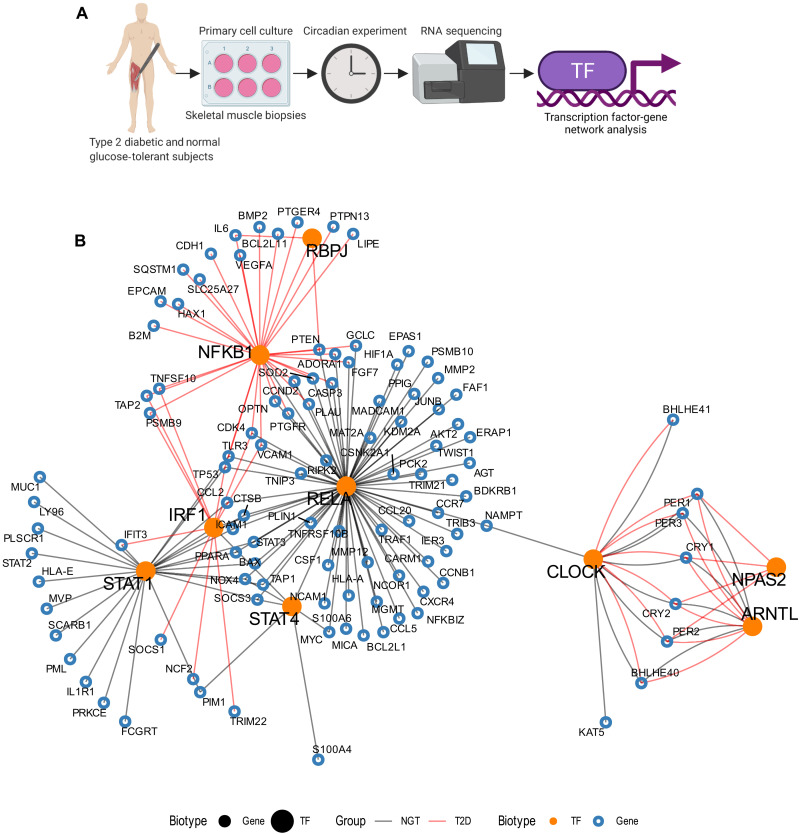
TF-gene interaction network analysis reveals divergent circadian TF enrichment of NPAS2 and RELA between T2D and NGT. (**A**) Schematic overview of primary cell culture circadian experiment and RNA sequencing. RNA sequencing of primary human skeletal muscle cells (myotubes) from men with NGT (*n* = 7) or T2D (*n* = 5). These data were used for the TF-gene network analysis. (**B**) The human TF-gene interactions were retrieved from TRRUST database version 2. Genes interacting with each TF were checked for enrichment with rhythmicity using Fisher’s exact test (FDR < 0.10). Large orange dots: TFs; small blue open circles: genes. Black and red lines represent the TF-gene interactions with rhythmic genes for myotubes from NGT and T2D donors, respectively.

To elucidate putative mechanisms driving altered circadian cross-talk of the inner-mitochondrial metabolism and clock-gene expression between myotubes from T2D and NGT donors, we performed transcription factor (TF)–gene network analysis on the circadian RNA-sequencing data presented in Fig. 1. This TF-gene network analysis revealed that genes interacting with CLOCK and ARNTL TFs were enriched for circadian genes, inferring a potential binding to these TFs in both the myotubes from NGT and T2D donors. However, NPAS2 was only enriched for circadian TF activity in myotubes from T2D donors. This finding is noteworthy since disruption of the mitochondrial inner membrane via OPA1 depletion increases *NPAS2* mRNA expression (Fig. 6). Outside of the core-clock TFs, a large cluster of TF-gene interactions was observed within the RELA proto-oncogene, NF-kB subunit (RELA) TF-associated gene network in NGT, but not T2D, while nuclear factor kappa B subunit 1 (NFKB1)-related TF-gene interactions were observed exclusively in myotubes from T2D donors (Fig. 7B). The circadian genes interacting with RELA, which was exclusively enriched in myocytes from NGT donors, included hypoxia-inducible factor-1α (*HIF1*α), which plays a role in oxygen sensing and circadian molecular signaling from the core clock to mitochondria (*30*).

### siRNA depletion of OPA1 increases mitochondrial reactive oxygen species, and OPA1-mediated changes in clock-gene expression can be restored by resveratrol treatment

**Fig. 8.**
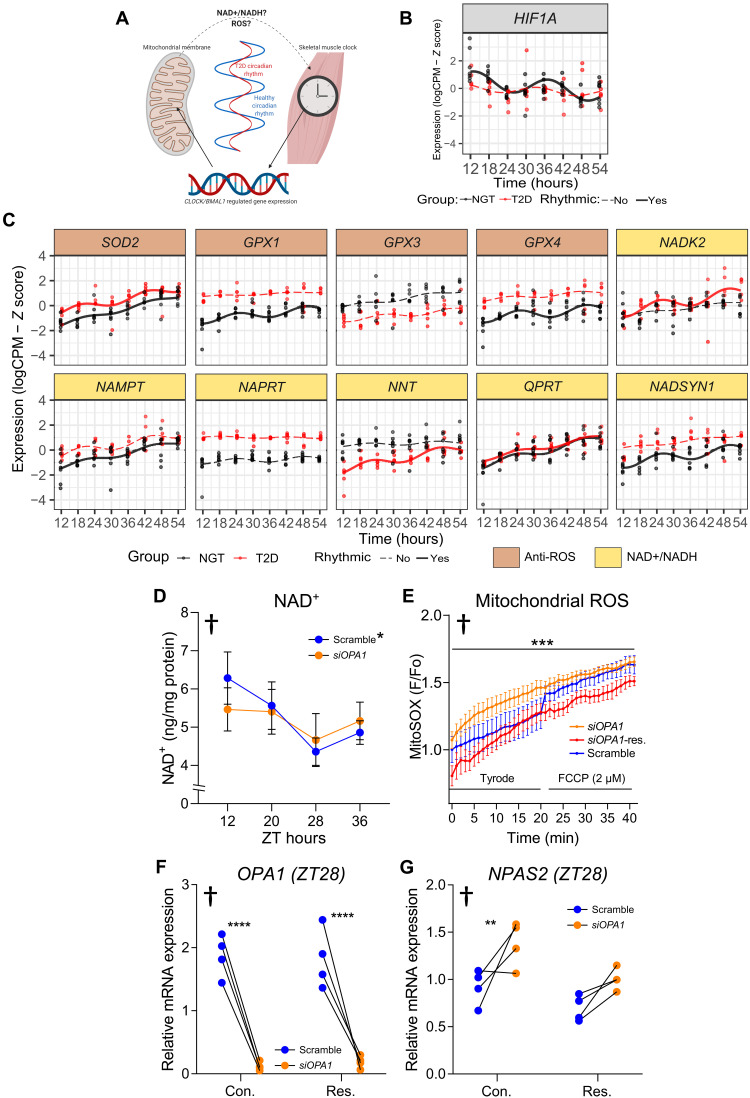
siRNA depletion of OPA1 increases mitochondrial ROS, and OPA1-mediated changes in clock-gene expression can be restored by resveratrol. (**A**) Circadian rhythms, transcription factor activity, and circadian inner-mitochondrial membrane metabolism are disrupted in T2D. Changes in NAD^+^/NADH metabolism and ROS signaling constitute a feedback loop permitting bidirectional control of circadian metabolism. (**B**) Time course of HIF1α mRNA in synchronized myotubes from donors with NGT (*n* = 7) or T2D (*n* = 5). Lines show harmonic regression fits, the solid line indicates circadian (FDR_RAIN_ < 0.1) genes, and dashed lines indicate non-circadian genes. (**C**) mRNA expression of antioxidant enzymes, and those linked to NAD^+^/NADH metabolism that localize to mitochondria in myotube cultures as described in (B). (**D**) NAD^+^ concentration in synchronized myotubes from NGT donors (*n* = 6) treated with siRNA against a scrambled sequence (blue) or *OPA1* (orange), two-way ANOVA, †overall time effect, *P < 0.05 difference between time points (12 versus 20 hours, and 28 versus 36 hours) only in Scramble. (**E**) Live-cell microscopy measuring MitoSOX fluorescence, indicative of mitochondrial ROS in myotube cultures as described in (D). Mixed-effects analysis, †*P* < 0.05 overall difference between conditions, ***P < 0.0001 difference between *siOPA1* versus *siOPA1*-resv, Scramble versus *siOPA1*, and Scramble versus *siOPA1*-resv. (**F**) *OPA1* and (**G**) *NPAS2* mRNA assessed at ZT28 in synchronized myotubes from NGT donors (*n* = 4) treated with siRNA against a scrambled sequence (blue) or *OPA1* (orange). Cells were incubated in the absence (vehicle control) or presence of 10 μM resveratrol. Relative to Scramble siRNA at ZT28. Two-way ANOVA, †*P* < 0.05 overall effect siRNA (Scramble versus *siOPA1*), **P < 0.01 control-Scramble versus control-treated *siOPA1*. ***P < 0.001 *siOPA1* versus Scramble. Results are mean ± SEM.

Our data suggest that the inner mitochondrion is a modulator of the molecular-clock genes *NPAS2*, *PER2*, and *PER3* in human skeletal muscle (Fig. 6), implicating retrograde signaling between OPA1 and the molecular clock. This signaling pathway may also be relevant for insulin sensitivity, since myotubes from T2D donors have altered circadian expression of mitochondrial genes and ablated rhythmic metabolism and a moderate alteration of some core-clock gene rhythms. Taking this into account, and given the data presented in Fig. 7, there are at least two plausible candidate pathways through which inner-mitochondrial–mediated signaling to the core clock may occur (Fig. 8A). First, nicotinamide adenine dinucleotide (NAD^+^) is a cofactor reflective of mitochondrial metabolism, and NAD^+^-dependent sirtuins are regulators of molecular-clock genes (*31*). Second, oxygen sensing plays a key role in molecular signaling from the core clock to mitochondria and vice versa in skeletal muscle via HIF1-α (*30*). *HIF1-*α mRNA displayed circadian rhythms in the myotube cultures from the NGT but not the T2D donors in our RNA-sequencing data (determined via RAIN Fig. 8B). HIF1-α expression is driven by hypoxia in skeletal muscle and regulated by reactive oxygen species (ROS) (*32*). Thus, we explored the circadian mRNA patterns within our RNA-sequencing data of myotubes from T2D and NGT donors to identify genes that localize to mitochondria, which are associated with ROS processing and NAD^+^/NADH (reduced form of NAD^+^) metabolism. Genes that were circadian in either T2D or NGT are highlighted in Fig. 8C, with antioxidant enzymes showing circadian regulation in NGT but not T2D (*GPX1* and *GPX4*). In addition, several genes involved in NAD^+^/NADH were circadian in NGT but not T2D (*NAMPT* and *NADSYN1*), while *NNT* was circadian in T2D but not NGT. Of note, *SOD2* (FDR = 0.0005) and *NAMPT* (FDR = 0.007) correlated with insulin sensitivity in our microarray data (Spearman’s correlation, fig. S6). Given the apparent rhythmic differences in these metabolic pathways, we next assessed the corresponding metabolites in response to internal mitochondrion dysregulation. Control cells from NGT donors had differences in NAD^+^ concentration over time, while cultures treated with siRNA targeting OPA1 (siOPA1) had an NAD^+^ concentration that did not differ over time. However, there was no difference in NAD^+^ concentration between siOPA1 and Scramble (Fig. 8D). We then assessed mitochondrial ROS levels using live-cell microscopy and measured MitoSOX fluorescence, indicative of mitochondrial ROS. OPA1-depleted myotubes had increased MitoSOX fluorescence when perfused with Tyrode’s solution, as compared to Scramble control (Fig. 8E). We also treated OPA1-depleted myotubes with resveratrol. Resveratrol can act as an antioxidant and reduce HIF1-α activity, in addition to increasing SIRT1 activity (*33*). Resveratrol reduced mitochondrial ROS levels in OPA1-depleted myotubes and rescued the OPA1-mediated changes in *NPAS2* expression (Fig. 8, F and G). Our results provide evidence to suggest that OPA1 regulation of mitochondrial ROS levels constitute a feedback loop permitting bidirectional control of circadian metabolism in skeletal muscle.

## DISCUSSION

Defective mitochondrial function and content is implicated in skeletal muscle insulin resistance and T2D pathogenesis (*24*, *34*, *35*). Our data provide mechanistic insight into this metabolic dysfunction and show that myotubes from individuals with T2D have an intrinsically disrupted circadian rhythm at the global transcriptomic and molecular-clock–specific level. Furthermore, an acute treatment of myotubes with a high concentration of glucose and insulin, partially mimicking a diabetic milieu, somewhat replicates the reduced number of rhythmic genes in T2D. However, clear differences between T2D and treatment with high glucose and insulin concentrations exist, including the number, percentage, and cellular location of genes at each peak time. These data suggest an intrinsic conservation of the T2D circadian signature in primary myocytes. This finding is consistent with data demonstrating that induced pluripotent stem cells from donors with T2D that were differentiated into myoblasts had multiple defects, including reduced insulin-stimulated glucose uptake and reduced mitochondrial oxidation (*36*). These T2D-associated defects were conserved despite the robust manipulations that the cells undergo. How cultured myocytes from T2D donors preserve a dysfunctional phenotype, including insulin resistance (*37*, *38*), has not been fully elucidated, but likely reflects genetic background and epigenetic mechanisms. Our data reveal that an intrinsic disruption of circadian biology in skeletal muscle may also be present in individuals with T2D. The cause of this disruption is unknown, but in addition to inherited factors, glucoregulatory disturbances, cumulative effects of sedentary lifestyle, sleep deprivation, and nutritional and hormonal factors are likely to play a role (*39*, *40*). Thus, genetic/epigenetic components and environmentally driven factors affect the circadian biology of metabolism.

Our results demonstrate that dysregulated rhythmic mitochondrial metabolism may play a role in mediating the disrupted rhythmic cellular metabolism and molecular-clock machinery in primary myotubes from individuals with T2D. Mitochondrial diurnal rhythms in oxidative capacity and oxygen metabolism have been demonstrated in several tissues in vivo, including skeletal muscle (*13*). Skeletal muscle diurnal mitochondrial rhythms are more likely to emanate from mitochondrial membrane dynamic processes, regulated by rhythmic proteins, such as OPA1 and FIS1, rather than a day-night rhythm of mitochondrial biogenesis and content (*26*, *41*). Mitochondrial membrane dynamics also demonstrate circadian behavior in several peripheral tissues (*41*); however, transcriptional control of these pathways is tissue specific (*35*, *42*). For example, while *Drp1*, *Mfn1/2*, and *Opa1* are not direct targets of the clock machinery in liver (*43*), *Mfn1* and *Opa1* are decreased in cardiac muscle of *Bmal1^−/−^* mice (*44*).

When assessing functional differences in the OCR over a 16-hour period, we observed differential rhythmicity between the myotubes from the NGT and T2D donors, with a lack of rhythmicity in T2D. The lack of a 24-hour rhythm in OCR in myotubes from the NGT and T2D donors is perplexing, but these cells are cultured in the absence of physiological or environmental cues, which may play a role. In addition, the serum shock cell-culture conditions were optimized to synchronize rhythmicity of circadian genes. Thus, we cannot exclude the possibility that the OCR rhythm in primary cells has a less robust 24-hour cycle as compared with core-clock gene expression. We speculate that OCR and mitochondrial function, which display changes over time in a diurnal manner in human skeletal muscle in vivo (*26*), maintain a physiological diurnal rhythm that can be fine-tuned in response to external stimuli, such as nutrient intake and physical activity.

In the current study, ChIP sequencing revealed that inner-mitochondrial cellular compartments were enriched for genes highly associated with insulin sensitivity, which were also direct targets of both CLOCK and BMAL1 in skeletal muscle. Few of these genes were also circadian in myotube cultures from donors with T2D when treated with high concentrations of glucose and insulin. Furthermore, few genes with CLOCK and BMAL1 binding coalesced with inner-mitochondrial genes in myotubes from donors with NGT, despite the similar enrichment of these pathways when assessing cycling genes with peaks at ZT24 and genes that correlated with insulin sensitivity in vivo. Metabolic pathways are independently capable of maintaining circadian rhythms in vitro (*45*), and our data suggest that maintenance of circadian mitochondrial metabolism in NGT may be partly independent of genes with core-clock binding.

Mitochondrial metabolism plays a role in bidirectional regulation of diurnal metabolism in peripheral tissues (*46*). Here, we report that myotube cultures from donors with NGT displayed more robust mitochondrial metabolic rhythms as compared with T2D. Mitochondrial rhythms in individuals with NGT may help to maintain the diurnal metabolic rhythm in skeletal muscle. In addition, manipulating mitochondrial membrane metabolism may alter the rhythmic expression of molecular-clock genes (*16*, *21*), although the signaling mechanism between mitochondrial metabolism and the molecular clock has not been elucidated. Our results provide evidence that *OPA1* depletion disrupts molecular-clock genes *NPAS2, PER2*, and *PER3* in primary myocytes. Depletion of OPA1 through either constitutive loss in mouse skeletal muscle or transient siRNA-mediated silencing in primary human myotubes resulted in differing effects on some core-clock genes. Of note, *Opa1^−/−^* mice have a myopathic phenotype (*23*), which may contribute to these differences. Collectively, we provide evidence that manipulating inner-mitochondrial metabolism alters *mRNA* expression of clock genes in skeletal muscle.

Mitochondrial respiration and ROS production are tightly linked, and alongside mitochondrial capacity and oxygen consumption, antioxidant proteins and ROS production also display circadian activity (*41*). In our study, mRNA expression of antioxidant enzymes that localize to mitochondria (*GPX1* and *GPX4*) were rhythmic in myotube cultures from donors with NGT, but not T2D. The aforementioned enzymes are involved in the detoxification of hydrogen peroxide, a known regulator of HIF1-α activity (*32*), which was also rhythmic in myotube cultures from donors with NGT, but not T2D. In addition, TF-gene network analysis demonstrated divergent circadian TF enrichment of NFKB1 and RELA between T2D and NGT. Both NFKB1 and RELA are subunits of nuclear factor kappa-light-chain-enhancer of activated B cells, which is highly responsive to ROS (*47*). Moreover, the circadian genes interacting with RELA, which was exclusively enriched in myocytes from NGT donors, included *HIF1*α. HIF1-α plays a key role in mediating signaling from the core clock to mitochondria and vice versa in skeletal muscle (*30*). Our data indicate that altered rhythms of mitochondrial respiration and ROS handling in myotube cultures from donors with T2D may be partly responsible for the ablated rhythm of HIF1-α expression. In support of this, the OPA1-mediated increase in *NPAS2* expression and mitochondrial ROS were rescued by treatment with resveratrol, an antioxidant and HIF1-α modulator. Collectively, our data show cross-talk between the inner mitochondrion and the molecular clock, with genes of these pathways rhythmically dysregulated in T2D. Dysfunction of the inner mitochondrion in peripheral tissues is implicated in the pathogenesis of insulin resistance and T2D, concomitant with increased ROS production, decreased oxidative capacity, and reduced metabolic flexibility (*35*). Thus, disturbances in daily rhythms of mitochondrial activity may contribute to skeletal muscle insulin resistance in T2D.

In conclusion, we show disturbances in the intrinsic rhythmicity of gene expression and metabolism in skeletal muscle cells of individuals with T2D. We find that the core molecular clock machinery is still largely intact in T2D, indicating that the major impact of diabetes is on the clock output, such that daily rhythms of metabolic and other genes are disturbed. This dysregulation is evident in myocytes in the absence of systemic factors, hormones, nutritional cues, or direct influence of the central or other peripheral clocks. Moreover, inner mitochondrion–associated genes displayed impaired cycling behavior in T2D. These inner-mitochondrion genes are also associated with insulin sensitivity and are under bidirectional regulation of circadian metabolism in skeletal muscle. The dysregulation of circadian metabolism in skeletal muscle of people with T2D underscores the need to take circadian biology into account and consider approaches in chronomedicine (*48*) when prescribing pharmacological therapy, particularly treatments that affect mitochondrial function. Our findings provide mechanistic insight into T2D pathophysiology and have clinical implications into the link between insulin sensitivity and environmental triggers that are associated with altered metabolism, including impaired sleeping patterns, social jet lag, or shift-work.

## MATERIALS AND METHODS

### Human study methods

#### Study group and skeletal muscle biopsy procedure

Study groups included individuals with NGT, as determined by oral glucose tolerance test OGTT, or T2D. Written informed consent was obtained from all participants. The study protocol was approved by the regional ethics board in Stockholm, Sweden. The participants were instructed to refrain from physical exercise 48 hours before the visit. All investigations were performed in the morning after an overnight fast. Anthropometric measurements were taken, and blood samples were obtained for clinical chemistry analysis. After application of local anesthesia (10 mg/ml; mepivacaine hydrochloride, AstraZeneca, Cambridge, UK), a biopsy was obtained from m. vastus lateralis using a Weil-Blakesley conchotome instrument (Agnthos, Sweden). Biopsies were cleared from any visible fat, connective tissue, or blood vessels and either used directly to prepare primary muscle cell cultures or immediately frozen in liquid nitrogen and stored at −80°C until microarray analysis. The clinical characteristics of the NGT (*n* = 7) or T2D (*n* = 5) donors for the muscle cell culture studies are presented in table S4.

### Cell culture

Primary myoblasts from skeletal muscle biopsies from men with NGT and T2D were grown in Dulbecco’s modified Eagle’s medium/nutrient mixture F-12 (DMEM/F12) + GlutaMAX with 16% fetal bovine serum (FBS) and 1% antibiotic-antimycotic (100×). Differentiation was induced at 80% confluence by incubating the cells for 4 to 5 days in fusion medium consisting of 76% DMEM GlutaMAX with glucose (25 mM), 20% M199 (5.5 mM), 2% Hepes, and 1% antibiotic-antimycotic (100×) with zinc sulfate (0.03 μg/ml) and vitamin B12 (1.4 mg/ml), with a further addition of apo-transferrin (100 μg/ml) and insulin (0.286 IU/ml) before use. Experiments were performed on differentiated myotubes after an additional 3 to 5 days of incubation in the same media containing 2% FBS (postfusion media).

### Circadian experiments

After postfusion, DMEM with glucose (5.5 mM) was used in the postfusion medium to create a low-glucose condition, or cells were treated with a high concentration of glucose and insulin (50 nM insulin and 25 mM glucose) before serum shock. After 22 hours, cells were synchronized by serum shock (media containing 50% FBS, 2 hours) (*49*) and then switched back to low-glucose media. Skeletal muscle myotubes from men with either T2D or NGT were lysed at 6-hour intervals from 12 to 54 hours. ZT was determined as time after synchronization.

### RNA sequencing

RNA was checked for quality using the Agilent RNA 600 nano kit and Bioanalyser instrument (Agilent Technologies, Santa Clara, CA). Aliquots of RNA (1 μg) were analyzed using the Illumina TruSeq Stranded Total RNA with Ribo-Zero Gold protocol (Illumina) as previously described (*50*, *51*). Ribosomal RNA was removed from the sample using 35 μl of rRNA removal beads (Illumina) on a magnetic plate followed by cleanup of the ribosomal-depleted RNA with 193 μl of Agencourt RNAClean XP beads (Beckman Coulter), 70% ethanol wash, and elution into 10 μl elution buffer (Illumina). The RNA sample was fragmented for 4 min at 94°C in Elute, Prime, Fragment High Mix (Illumina) and then subjected to first-strand cDNA synthesis with 1 μl of Superscript III reverse transcriptase (Life Technologies) per sample using a thermocycler programmed to 25°C for 10 min, 50°C for 15 min, and 70°C for 15 min. Second-strand cDNA was synthesized by addition of Second-Strand Marking Master Mix, and samples were incubated at 16°C for 60 min. Samples were subjected to another bead cleanup before A-tailing and ligation of adapters as per kit instructions (Illumina). Following a third bead cleanup, samples were enriched for DNA fragments by amplification using the Illumina polymerase chain reaction (PCR) Primer Cocktail and PCR Master Mix, subjected to 98°C for 30 min, followed by a predefined cycle (98°C for 10 s, 60°C for 30 s, and 72°C for 30 s) that was repeated 3 to 15 times, on the basis of each individual sample, and finally incubated for 5 min at 72°C. Samples were cleaned and validated for DNA concentration using the Qubit dsDNA HS assay kit (Invitrogen) and for base pair size and purity using the Agilent High Sensitivity DNA chip and Bioanalyzer instrument. Libraries were subjected to 100-bp single-end sequencing on the X Ten platform (Illumina) at the Beijing Genomics Institute, Hong Kong, China. Approximately 20 million reads per sample were assigned to genes with 18,482 genes surviving the expression threshold.

### RNA-sequencing data analysis

RNA-sequencing reads ($\bar{n}$ ≈ 40 M) from FASTQ files were quality-trimmed using Trim_Galore (v0.4.3). Trimmed reads were aligned using STAR (v2.5.3a) (*52*) aligner with Ensembl human annotation (GRCh38, release 92) (*53*), and gene features were counted using featureCounts from subread (v1.5.2) package (*54*) resulting in 27 M uniquely mapped and 20 M assigned reads to genomic features (genes) on average, respectively. The lowly expressed genes were discarded from downstream analysis using filterByExpr function from edgeR package (*55*) resulting in 18,482 genes. As an apparent batch effect was introduced by participants (fig. S1), this batch effect was removed by using limma’s removeBatchEffect function (*56*) for further downstream rhythmicity (RAIN) and differential rhythmicity (DODR) analysis as these tools do not consider any batch effect by default. The batch-corrected counts per million (CPM) values were in log_2_ scale.

### Rhythmicity and differential rhythmicity analysis

*P* values for rhythmicity were assessed using RAIN (*17*) with longitudinal method and adjusted for multiple testing using the Benjamini-Hochberg method. Genes with adjusted *P* value below 0.10 (FDR_RAIN_ < 0.10) were considered as rhythmic. We chose RAIN because this algorithm detects both symmetric and nonsymmetric wave forms (*17*). To compare two groups for differential rhythmicity analysis, subsetting genes with rhythmic behavior is essential as comparing genes that have no rhythmic properties can result in false positives. Therefore, meta *P* values using a beta-distribution–based approach (the same as what DODR uses internally) from both conditions were calculated and adjusted for multiple testing using the Benjamini-Hochberg method. The genes with adjusted meta *P* values below 0.10 were subsetted for the subsequent differential rhythmicity analysis. Differential rhythmicity analysis was performed on mean-centered logCPM values (batch-corrected) between myotubes from NGT and T2D donors (control and HGI group, separately) by using DODR (*57*) with “robust” method and resulting meta *P* values were adjusted for multiple testing using the Benjamini-Hochberg method. It is essential to mean-center the logCPM values as DODR internally assumes identical means to test for differential rhythmicity while setting the normalization parameter to false. Genes with adjusted meta *P* value below 0.10 (FDR_DODR_ < 0.10) were considered differentially rhythmic.

### Circadian amplitude estimations

A harmonic regression model using HarmonicRegression R package (*58*) with first-degree polynomial component (linear regression) was fit to (participant) batch-corrected logCPM values. As relative amplitudes are dimensionless compared to absolute amplitudes, it allows for a proper comparison across different conditions and experiments while normalizing for systematic errors (*59*). Since our batch effect (from individuals)–corrected RNA expression values were in log_2_ scale (logCPM), we needed to perform appropriate transformations to calculate the relative amplitudes. The fold-change amplitude (*A*_fc_) is defined as the ratio between the peak (*e*_max_) and the trough (*e*_min_) values

$$A_{\text{fc}}=\frac{e_{\text{max}}}{e_{\text{min}}}$$ (1)

Twice the absolute amplitude calculated from log_2_-transformed data (*A*_log_) is equal to log_2_ of fold-change amplitude (*A*_fc_)

$${\text{log}}_{2}(A_{\text{fc}})={\text{log}}_{2}(e_{\text{max}})-{\text{log}}_{2}(e_{\text{min}})=2.A_{\text{log}}$$ (2)

$$2.A_{\text{log}}={\text{log}}_{2}(A_{\text{fc}})$$

Considering the relationship between the fold-change amplitude and the relative amplitude (*59*)

$$A_{\text{fc}}=\frac{1+A_{\text{rel}}}{1-A_{\text{rel}}}$$ (3)

The relative amplitude (*A*_rel_) can be derived by solving Eqs. 2 and 3 to explain the relationship between *A*_rel_ and *A*_log_ as

$$A_{\text{rel}}=\frac{2^{2.A_{\text{log}}}-1}{2^{2.A_{\text{log}}}+1}$$ (4)

To compare the relative amplitudes between T2Ds and NGTs, log_2_ ratios were calculated for each treatment group (control and HGI)

$${\text{log}}_{2}(A_{\text{rel}})={\text{log}}_{2}(\frac{A_{{\text{rel}}_{T2D}}}{A_{{\mathrm{rel}}_{\text{NGT}}}})$$ (5)

The log_2_(*A*_rel_) of circadian genes was compared to log_2_(*A*_rel_) of noncircadian genes using a two-sided Kolmogorov-Smirnov test (Fig. 2A). Individual relative amplitudes of circadian genes of NGT and T2D groups were compared to each other using paired Wilcoxon rank sum test (Fig. 2B).

### Heatmaps for the circadian gene expression

The RNA-sequencing gene expression in our cohort had an increasing or decreasing linear trend over time. Therefore, we have fitted a harmonic regression model with first-degree polynomial to our data to show the rhythmic patterns for each gene

$$E=a.\text{sin}(\frac{2\pi }{w}t)+b.\text{sin}(\frac{2\pi }{w}t)+(c+d.t)$$ (6)

where *E* is the fitted expression value, *w* is the period (24 hours), *t* is the time in hours, and *a*, *b* and *c*, *d* are the coefficients for the harmonic regression and linear regression, respectively. However, visualization of the rhythmic data with a linear trend on a heatmap is problematic owing to the extreme values masking the peak and trough values. Therefore, we have removed the linear trend from our data by fitting a linear line and subtracting it from the previously fitted harmonic regression with linear trends

E′=E−(α+β.t) (7)

where *E*′ is the fitted expression values without linear trends and α and β are the coefficients for the linear regression. Before linear trend removal, expression data are mean-centered or *z* score–transformed across time points for each NGT and T2D group to show the relative amplitude differences (Fig. 2B) or rhythmic patterns (fig. S2), respectively.

### Gene enrichment analysis

Gene enrichment analysis was performed by using ORA throughout this study. For biological interpretation of the biological processes, we used Reactome pathways (*19*), while GO (*60*) CC were used to identify the enrichment of subcellular compartments. clusterProfiler (*61*) R package was used to analyze all gene enrichment results. Ontology terms (Reactome, GO:CC) with an adjusted *P* value, using the Benjamini-Hochberg method, below 0.10 were considered to be significantly enriched.

As we aimed to investigate the biological pathways and CC associated with the circadian genes, we subsetted circadian genes by disease-treatment groups (Fig. 1D) and peak times (Fig. 3F) and analyzed enrichment from Reactome pathways and GO:CC using ORA, respectively. Similarly, genes with significant correlations between insulin resistance (*M* value) and basal gene expression (Fig. 4C) were tested for GO:CC enrichment using the ORA method. The top 10 enriched terms ranked by gene ratio (the number of significant genes associated with the given the ontology term divided by all significant genes that are a member of any ontology term) were plotted in Figs. 1D, 3F, 4C, and 5C. An ontology-gene interaction network with the top five enriched (ORA) GO:CC terms ranked by gene ratio (the so-called CNET plot) was generated to show the involvement of an individual gene, together with the Spearman correlation coefficient (Fig. 4D).

Last, clustering analysis revealed 12 distinct clusters from integrative analysis using binary input for the presence/absence of rhythmicity in disease-treatment groups, insulin sensitivity correlation with basal gene expression, and BMAL/CLOCK binding. The genes in each cluster were analyzed using the GO:CC database with the ORA method (Fig. 5C), similar to the analysis performed in Figs. 1D and 3F.

#### Hyperinsulinemic-euglycemic clamp procedure

This study group was handled as described in the “Human study methods” section. The clinical characteristics of the men with NGT (*n* = 24) or T2D (*n* = 25) enrolled in the gene array and hyperinsulinemic-euglycemic clamp study are reported in table S5. After a 45-min rest, the study participants underwent a hyperinsulinemic-euglycemic clamp as previously described (*62*). Following an intravenous bolus dose of insulin (1600 mU/m^2^ body surface area), insulin was infused intravenously at a rate of 40 mU/m^2^ per minute for 2 hours, and a variable intravenous infusion of glucose (200 mg/ml) was used to maintain euglycemia between 81 and 99 mg/dl (4.5 to 5.5 mM). The infusion rate of glucose during the last 60 min of the clamp, when insulin levels are in a steady state, was used to calculate whole-body glucose disposal rates (*M* value).

### ChIP sequencing

#### Tissue harvesting

Animal procedures were conducted in accordance with institutional guidelines for the care and use of laboratory animals as approved by the University of Florida Institutional Animal Care and Use Committee. For the ChIP-sequencing data, two replicate samples for BMAL1 and CLOCK were used. Each sample required pooling gastrocnemius muscle from 10 adult C57BL/6J male mice (the Jackson Laboratory, Farmington, CT, USA). The mice were entrained to a 12-hour light/12-hour dark schedule, and all tissues were collected at ZT2 and frozen immediately.

#### ChIP-sequencing sample preparation

For the CLOCK ChIP-sequencing samples, skeletal muscle was homogenized (muscle:buffer ratio, 1:10) in lysis buffer [10 mM Hepes (pH 7.5), 10 mM MgCl_2_, 60 mM KCl, 300 mM sucrose, 0.1 mM EDTA (pH 8.0), 0.1% Triton X-100, and 1 mM dithiothreitol (DTT)] with ethylene glycol-bis (succininic acid) *N*-hydroxysuccinimide ester (Bio-world) (*63*). The reaction was stopped with 1 M tris (pH 7.5) buffer (final concentration of tris was 20 mM), and formaldehyde was added (final concentration of 1%). Samples were incubated for 25 min at room temperature, and cross-linking was stopped by adding glycine to 125 mM.

For the BMAL1 ChIP-sequencing samples, we followed a protocol outlined previously (*64*). Skeletal muscles were homogenized in lysis buffer containing 1% of formaldehyde. Samples were incubated at room temperature for 25 min, and cross-linking was stopped by adding glycine to 125 mM.

For all samples, myonuclei were isolated from skeletal muscle homogenates as previously described (*64*), and samples were incubated with the appropriate antibody at 4°C overnight. Samples were washed and then incubated at 65°C overnight to decross-link. Last, ribonuclease, 5 M NaCl, and proteinase K were added, and samples were incubated at 55°C for 1 hour.

DNA was recovered with a PCR purification kit (Qiagen), and the ChIP-sequencing library was prepared by using NEBNext Ultra DNA library prep kit for Illumina. Library quality was determined by Tapestation and quantitative PCR. Sequencing parameters were paired-end 100 bp run with approximately 40 M reads per sample on the HiSeq 3000 Illumina sequencing system.

#### ChIP-sequencing data analysis

The reads were aligned to *Mus musculus* genome assembly GRCm38 (mm 10) using Bowtie2 (*65*) with the --sensitive-local option, which does not require that the entire read aligns from one end to the other. The biological replicates of the aligned reads were merged for *CLOCK*, *BMAL1*, and input, respectively. Homer software (*66*) was deployed to perform peak calling for the CLOCK sample and the BMAL1 sample with the input sample as background. The default FDR rate threshold 0.001 was used to detect significant peaks.

### Integrative analysis of circadian rhythmicity, insulin sensitivity, and BMAL1/CLOCK chromatin binding

To identify the gene patterns across different experimental procedures, an integrative analysis approach was applied to circadian genes (RNA sequencing), insulin sensitivity correlations (*M* value versus basal gene expression from microarray study), and BMAL1/CLOCK chromatin binding (ChIP sequencing). Both gene expression correlations with *M* values and BMAL1/CLOCK ChIP-sequencing experiments were performed on mice; therefore, we have identified the homologous genes between mouse and human and only included those genes for further downstream analysis. ChIP-sequencing peaks close to a gene’s TSS with maximum peak score are assigned to that gene. A binary matrix was created with the circadian rhythmicity (1: rhythmic, 0: nonrhythmic), Spearman correlation between insulin sensitivity (*M* value) and basal gene expression (1: significant, 0: nonsignificant), and BMAL1/CLOCK binding (1: bound, 0: not bound). The resulting matrix was used for clustering analysis using the “clust” algorithm (*67*) to identify groups of genes showing similar patterns. This resulted in 12 different clusters. Those 12 clusters were used for gene enrichment analysis using the ORA method and the GO:CC database.

The circadian genes associated with BMAL1/CLOCK binding were identified by overlapping each group, NGTs and T2Ds, with both treatment conditions separately. The overlaps were tested by Fisher’s exact test, and the resulting *P* values were adjusted by the Benjamini-Hochberg method. Adjusted *P* values below 0.05 were considered as statistically significant. Overlapping gene percentage, also known as Jaccard index, was used for showing the similarity between the overlaps (Fig. 5A).

### TF-gene interaction network of rhythmic genes

The TF-gene interactions for human were retrieved from TRRUST database version 2 (*68*). The nature of the interaction (activation, inactivation, and unknown) was omitted because of its complexity and experimental setup differences. Since all gene IDs were presented as gene symbols/names, we converted all gene symbol IDs to Ensembl IDs to conform to our results, considering that the same gene symbol ID can match with several Ensembl IDs. Genes interacting with each TF were extracted and checked for enrichment for NGT and T2D rhythmic genes, separately, using Fisher’s exact test (alternative hypothesis: the OR is greater than 1). Genes with no overlap between rhythmic genes and TF-gene pairs were excluded from the analysis. In total, 384 TFs were tested for enrichment with rhythmic genes in both NGT and T2D groups. *P* values were adjusted for multiple testing using the Benjamini-Hochberg method (*69*) for the NGT and T2D groups, separately. Enrichments with adjusted *P* value below 0.10 were considered significant.

### Animal models

#### Myo-Cre Opa1^−/−^ mice

Animal work was performed in compliance with guidelines established by the University of Barcelona Committee on Animal Care. *Opa1^loxP/loxP^* mice were generated as previously reported (*23*, *70*). The *Myo-Cre OPA1* (Opa1 mKO) mouse line was generated by crossing homozygous *OPA1^loxP/loxP^* mice with a strain expressing *Cre* recombinase under the control of the myogenin promoter and the 1-kb mouse myocyte enhancer factor 2, thus yielding a transgene called *Myo*-*Cr*e (*71*). *Cre OPA1* littermates were used as controls for knockout mice. Mice were kept under a 12-hour dark/light cycle and were provided standard chow diet and water ad libitum. Four-month-old male mice were used in all experiments. On the experimental day, mice were anesthetized with isoflurane, euthanized by cervical dislocation, and tibialis anterior muscles were harvested and immediately frozen in liquid nitrogen for subsequent analysis.

### Mitochondrial-disrupting compounds and circadian studies

Primary human skeletal muscle cells from men with NGT were used to study the effect of compounds FCCP, rotenone A, antimycin A, and oligomycin on molecular-clock gene expression. Primary skeletal muscle cells were grown and differentiated as described above. The cells were synchronized as described above, compounds or vehicle control were added at ZT14 after synchronization, and cells were harvested after 4-hour exposure (ZT18). Compounds were rotenone/antimycin A (0.38 μm), FCCP (2 μm), and oligomycin (1 μm). Cells were harvested to collect RNA, and core-clock gene mRNA expression was measured by quantitative reverse transcription PCR. A lactate dehydrogenase (LDH) assay (no. 11644793001, Roche, Basel, Switzerland) was performed according to the manufacturer’s instructions to determine cell viability after treatment with compounds. Cell supernatant was collected when harvesting RNA; 100 μl of this supernatant was added to a 96-well plate, along with 100 μl of test solution (2.2% lyophilizate catalyst solution +97.8% dye solution) and incubated at room temperature for 30 min. Absorbance was measured at 490 and 690 nm (for background), and LDH content for each sample was calculated as OD_490_ (optical density at 490 nm) − OD_690_, presented as percentage relative to control. There were no differences in LDH concentration in any condition (fig. S7).

### Gene silencing and metabolic studies

#### Opa1 silencing

Primary human skeletal muscle cells from men with NGT were used for the study of *OPA1* silencing. Primary skeletal muscle cells were grown and differentiated as described above. After switching to postfusion media, cells were transfected as previously described (*21*), using 10 μM of either Silencer Select (Thermo Fisher Scientific, Waltham, MA) Negative Control No. 2 (no. 4390847) or validated siRNAs against *OPA1* (no. s9852), for two separate 5-hour transfection periods separated by ~48 hours.

#### Metabolic phenotyping of cells

To assess the mitochondrial function of primary human skeletal muscle cells, myotubes were subjected to a Seahorse XF Mito Stress Test using the manufacturer’s instructions for timing (Agilent, Santa Clara, CA) as previously described (*21*). OCR and extracellular acidification rates (ECARs) were measured at three time points under unstimulated conditions and then after treatment with 1 μM oligomycin, 2 μM FCCP, and 0.75 μM rotenone + antimycin A. OCR and ECAR were normalized to the protein concentration, as quantified by a Pierce bicinchoninic acid (BCA) protein assay kit (Thermo Fisher Scientific). Myotube cultures either from men with NGT (*n* = 5) or from those with T2D (*n* = 5) were subjected to a Mito Stress Test (fig. S1) under the same conditions as previously described, except these cells were measured at ZT24 after synchronization as described in the “Circadian experiments” section. For circadian Seahorse XF experiments (time course of basal OCR, Fig. 3H), myotube cultures from men with either NGT (*n* = 5) or T2D (*n* = 5) were synchronized as described in the “Circadian experiments” section. Myotubes were measured sequentially, i.e., the same cells were measured at all time points in buffered media, and only basal oxygen consumption was measured. This allowed minimal disturbance to cells and enabled measurements of diurnal oxygen consumption in a method derived from previous work (*31*).

#### NAD measurements of cells after OPA1 silencing

NAD levels were assessed in primary human skeletal muscle cells after *OPA1* silencing using an enzymatic cycling assay (*72*). Cells were harvested with trypsin, and the cell pellet was dissolved in 200 μl of 0.6 M perchloric acid and subjected to centrifugation for 3 min at 13,000*g*. The supernatants were transferred to new tubes and diluted 1:100 in 100 mM Na_2_HPO_4_ (pH 8). An aliquot (100 μl) of the diluted extract was pipetted into a 96-well plate, and a reaction mix (100 μl) containing 100 mM Na_2_HPO_4_, 10 μM flavin mononucleotide, 2% ethanol, alcohol dehydrogenase (90 U/ml), diaphorase (130 mU/ml), resazurin (2.5 μg/ml), and 10 mM nicotinamide was added. Fluorescence (excitation 540 nm/emission 580 nm) was measured over 30 min, and the NAD content was calculated from a standard curve and normalized to the protein concentration. Protein was extracted by dissolving the cell pellet (after perchloric acid precipitation) in 200 μl of 0.2 M NaOH, incubating at 95°C for 5 min, and centrifugation for 5 min at 13,000*g*. Protein concentration was determined on the supernatant using the BCA assay (23227, Thermo Fisher Scientific, MA).

### Resveratrol treatment

Human primary skeletal muscle cells were grown, differentiated, and synchronized as described above. Cells were treated with either 10 μM resveratrol (no. R5010, Merck KGaA, Darmstadt, Germany) or vehicle control for the duration of the experiment, subsequent to synchronization.

### Gene expression analysis

#### Microarray analysis

RNA quality was assessed and ensured using the Experion Automated Electrophoresis System (Bio-Rad Laboratories, CA). Affymetrix GeneChip Human Transcriptome Array 2.0 (Thermo Fisher Scientific, MA) was used for the whole-transcriptome analysis performed at the Bioinformatics and Expression Core facility at Karolinska Institutet, Huddinge, Sweden. Amplified and biotinylated sense-strand DNA targets were generated from total RNA using the WT Plus Kit (Thermo Fisher Scientific, MA). Fragmented and biotinylated sense-strand DNA target (5.5 μg) were hybridized to the arrays in GeneChip Hybridization Oven 645 at 45°C for 16 to 18 hours. Arrays were washed and stained in GeneChip Fluidics Station 450 before scanning in Affymetrix GeneChip Scanner.

Normalization and calculation of microarray (*n* = 49) gene expression was performed with the Robust Multichip Average expression measure using oligo R package (v1.56.0) (*73*). Probeset IDs were matched to their corresponding Ensembl IDs using biomaRt R package (v2.48.2). The nonunique probesets mapping to multiple Ensembl IDs were filtered out, and the probesets with signal value below 4 in at least 24 samples (minimum “Disease” group size) are discarded from the analysis. The remaining probeset expression values are collapsed to Ensembl IDs using collapseRows function (*74*) from WGCNA R package (v1.70-3) with the “MaxMean” method (*75*). The resulting expression matrix with Ensembl IDs (*n* = 13,570) were used for downstream analysis. The correlation between the basal gene expression and insulin sensitivity (*M* value) was calculated by Spearman’s rank correlation, and *P* values are adjusted for multiple testing using the Benjamini-Hochberg method. The genes with significant correlations with *M* values (*P*_adj_ < 0.10, *n* = 640) were used for gene enrichment analysis using an over-representation method with the GO:CC database (see the “Gene enrichment analysis” section for details). The ontology-gene interaction network (the so-called CNET plot) from this analysis using the top five enriched terms ranked by gene ratio is shown in Fig. 4D. The color code represents the correlation coefficient (Spearman’s ρ) between gene expression and *M* values.

#### PCR analysis

RNA was prepared from human skeletal muscle biopsies and primary human skeletal muscle cells. Gene expression was determined by using Fast SYBR Green Master Mix (Thermo Fisher Scientific) and self-designed oligonucleotides (oligonucleotide sequences provided below) or predesigned TaqMan Gene Expression Assays (Thermo Fisher Scientific). SYBR human housekeeping: *B2M*, *GUSB*, *TBP*, and *RPLO*. TaqMan mouse housekeeping genes: *B2m* and *Gapdh*; human: *TBP* and *B2M.* Primer sequences and assay IDs are presented in table S6.

### Immunoblot analysis

Immunoblot analysis was performed as previously described (*21*) with antibodies against OPA1 (no. 80471) from Cell Signaling Technology, total OXPHOS (no. 110411) from Abcam (UK), and β-actin (no. A5441) from Sigma-Aldrich. Protein abundance was normalized to β-actin.

### Live-cell microscopy and determination of mitochondrial ROS

Primary human skeletal muscle cells from men with NGT were used for the determination of mitochondrial ROS by live-cell microscopy. Primary skeletal muscle cells were grown and differentiated as described above. MitoSOX (50 μg; M36008, Thermo Fisher Scientific) was dissolved in 50 μl of pluronic F127 (P6866, Invitrogen) and 50 μl of dimethyl sulfoxide to make the MitoSOX stock solution. MitoSOX stock solution (5 μl) was added to the media and incubated for 15 min at 37°C and 5% CO_2_. Thereafter, media from the cell dish were removed; the dish was mounted on the confocal microscope (Zeiss LSM710) and perfused with Tyrode solution (in mM): 121 NaCl, 5 KCl, 1.8 CaCl_2_, 0.4 NaH_2_PO_4_, 0.5 MgCl_2_, 24 NaHCO_3_, 0.1 EDTA, and 5.5 glucose. Cells were then imaged with 2-min intervals for 20 min and thereafter switched to Tyrode solution supplemented with FCCP (2 μM) for 20 min. All images were analyzed with ImageJ.
