# Supplementary material for: Disrupted circadian oscillations in type 2 diabetes are linked to altered rhythmic mitochondrial metabolism in skeletal muscle
Source: Sci Adv. 2021 Oct 20;7(43):eabi9654. doi: 10.1126/sciadv.abi9654 (PMC8528429; doi:10.1126/sciadv.abi9654)
Supplement: Supplementary file 1 — Figs. S1 to S7 Tables S4 to S6 [file sciadv.abi9654_sm.pdf]

Science Advances
AAAS

Supplementary Materials for

Disrupted circadian oscillations in type 2 diabetes are linked to altered rhythmic mitochondrial metabolism in skeletal muscle

Brendan M. Gabriel, Ali Altıntaş, Jonathon A. B. Smith, Laura Sardon-Puig, Xiping Zhang, Astrid L. Basse, Rhianna C. Laker, Hui Gao, Zhengye Liu, Lucile Dollet, Jonas T. Treebak, Antonio Zorzano, Zhiguang Huo, Mikael Rydén, Johanna T. Lanner, Karyn A. Esser, Romain Barrès, Nicolas J. Pillon, Anna Krook, Juleen R. Zierath*

*Corresponding author. Email: juleen.zierath@ki.se

Published 20 October 2021, *Sci. Adv.* **7**, eabi9654 (2021)
DOI: 10.1126/sciadv.abi9654

The PDF file includes:

Figs. S1 to S7
Tables S4 to S6

Other Supplementary Material for this manuscript includes the following:

Tables S1 to S3

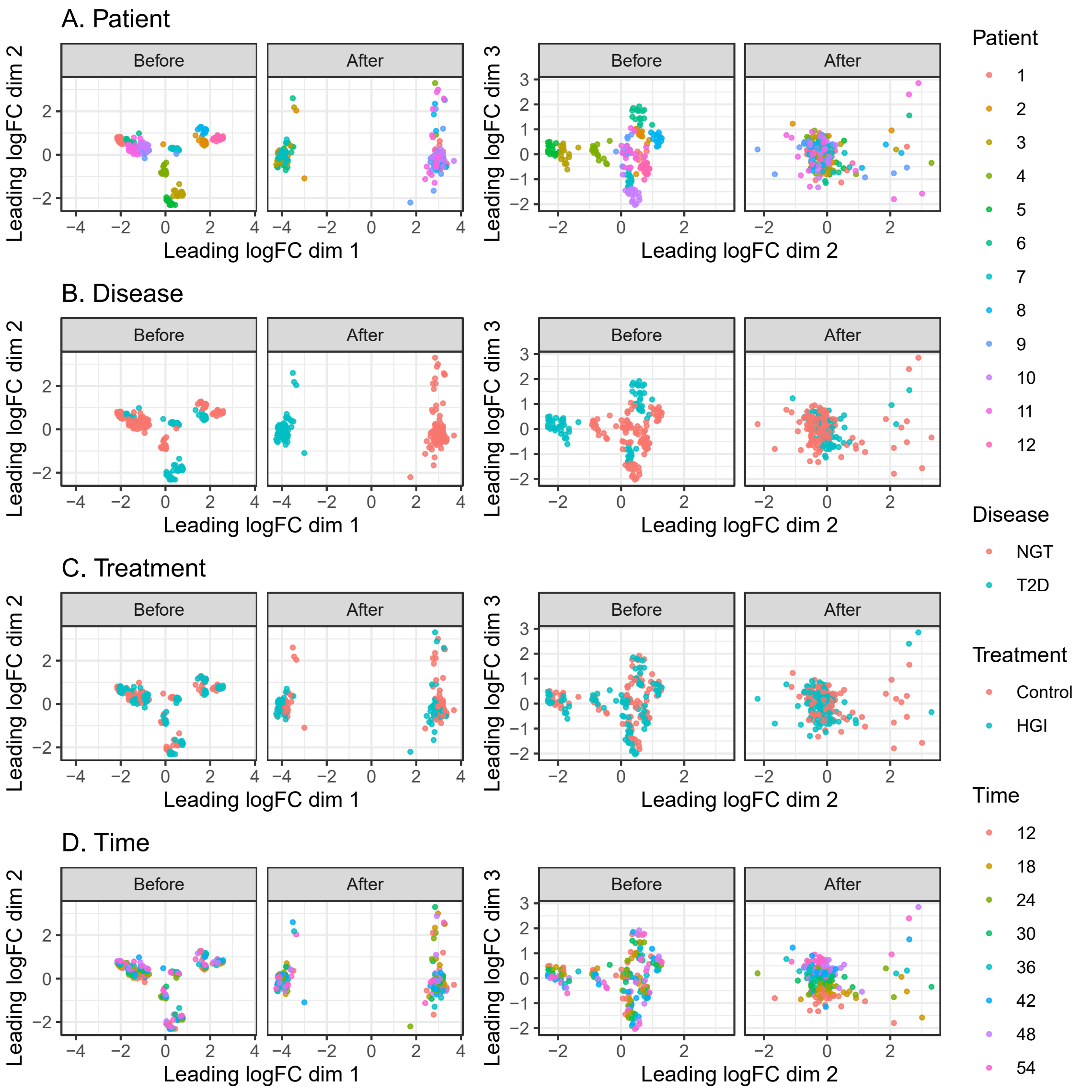

Figure S1. Multi-dimensional scaling (MDS) plots showing dimensions 1-2 (left panels) and 2-3 (right panels) before and after patient batch effect removal. Each experimental condition is presented in A) Patient, B) Disease, C) Treatment and D) Time. Dimension 1 separates the disease group while dimension 3 separates time of sampling after removing the patient batch effect. Time is ZT hours.

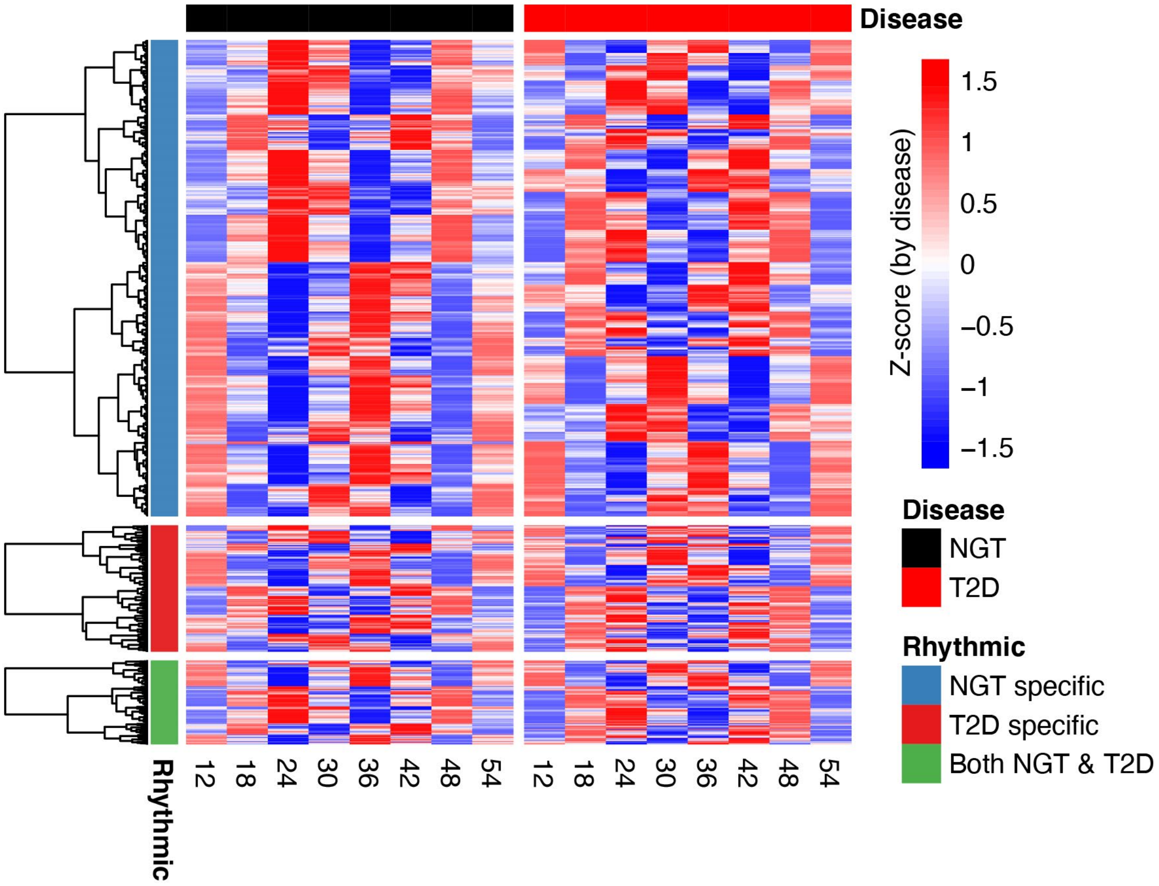

Figure S2. Heatmaps showing the rhythmic patterns of RNA-sequencing gene expression data of NGT and T2D groups. The linear trends are removed for visual purposes to highlight the patterns in data (see Methods section). To show the rhythmic patterns between NGT and T2D, the data is z-score transformed separately for the groups. Each time point shown in the heatmap is the mean value after z-score transformation. Hierarchical clustering was performed by using geodesic distance and 'ward.D2' algorithm separately for each subset of circadian genes in NGT and T2D groups.

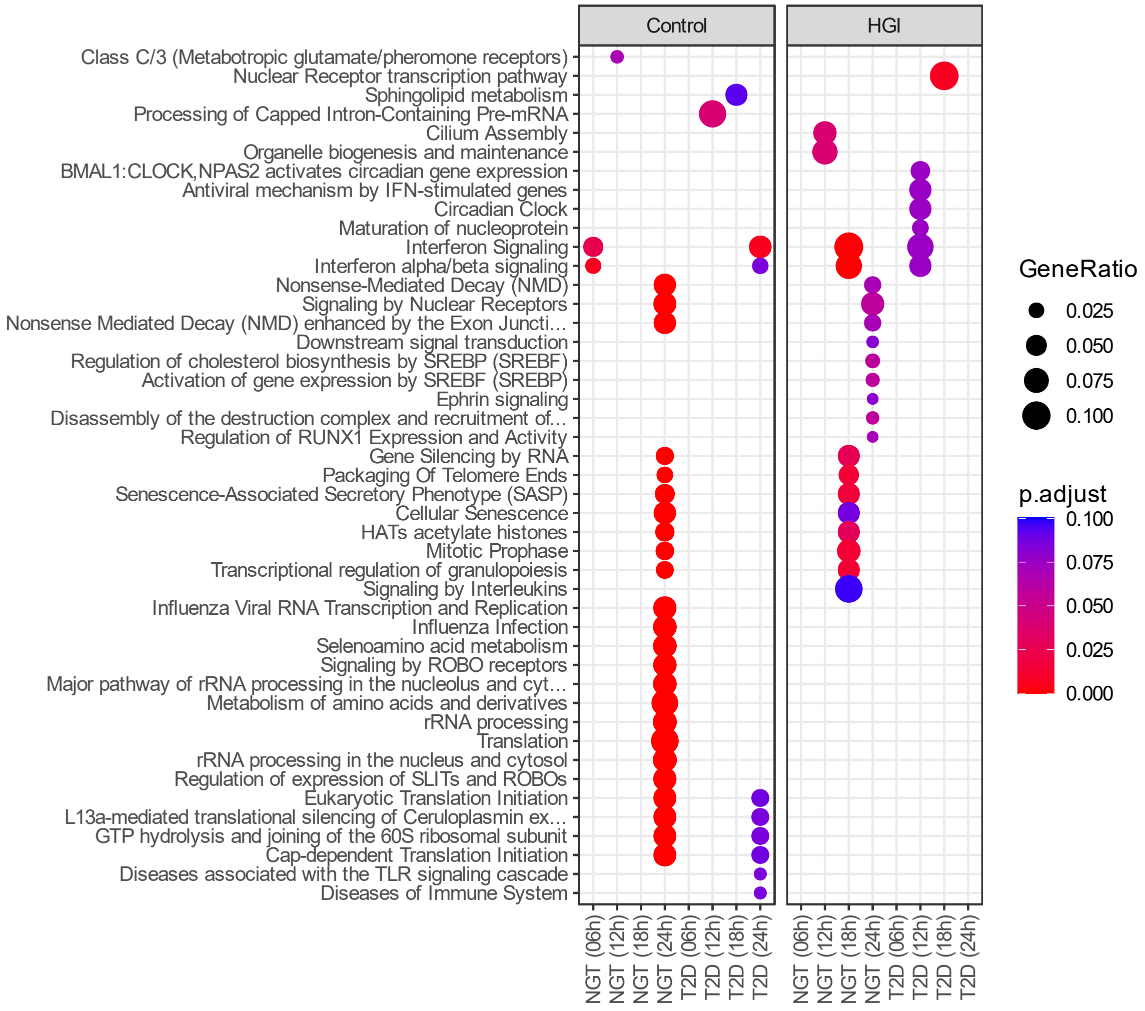

Figure S3. Reactome pathways enriched at each peak time in NGT (control), T2D (control), NGT (high concentration of glucose and insulin; HGI), and T2D (HGI).

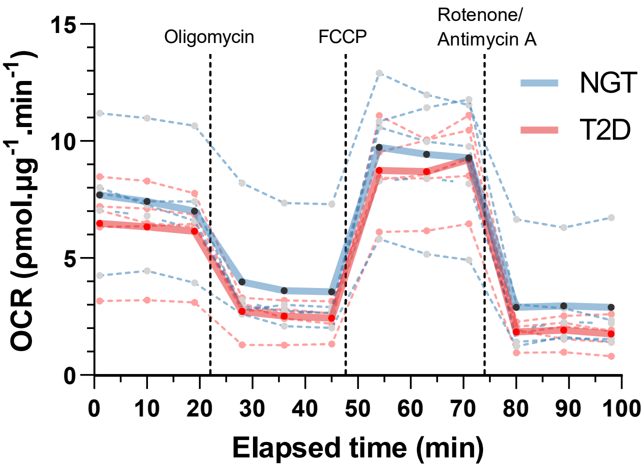

Figure S4. Mitochondrial respiratory function (Mito Stress test, Seahorse XF analyzer) at ZT24 in synchronized myotubes from donors with normal glucose tolerance (NGT; n=5) or Type 2 diabetes (T2D; n=5). 2-way repeated measures ANOVA of time-course. Solid lines are overall mean values, while dashed lines are means of individual donors.

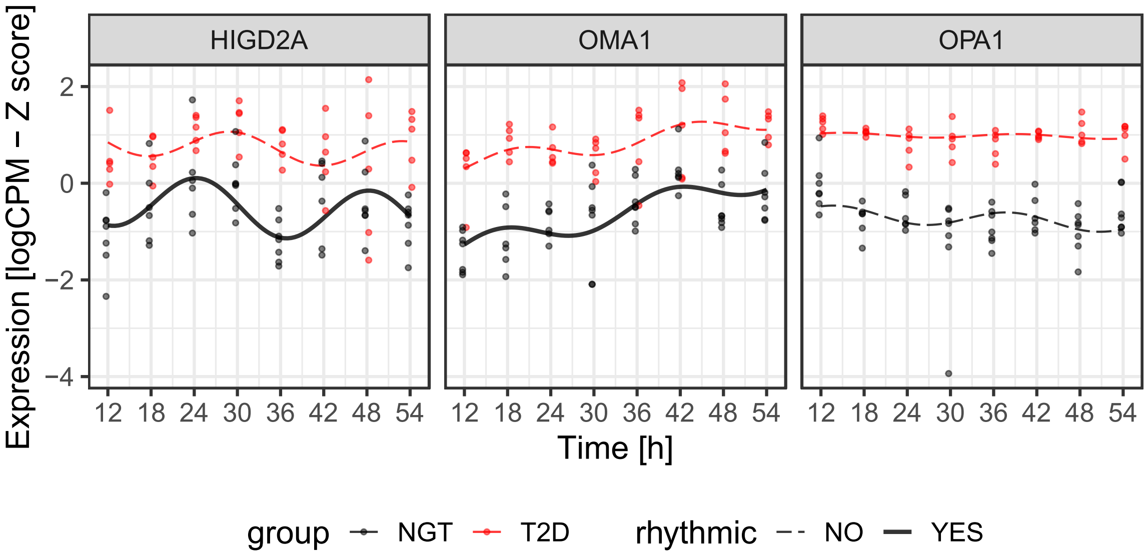

Figure S5. mRNA expression data from RNA sequencing of myotube cultures from donors with NGT or T2D (n=7, and 5, respectively) of *HIGD2A*, *OMA1*, and *OPA1*. Lines show the harmonic regression fits and solid line indicates circadian (FDR_{RAIN}<0.1) genes while dashed lines indicate non-circadian genes (RAIN analysis). Time points are hours post-synchronization. Black=NGT, Red=T2D.

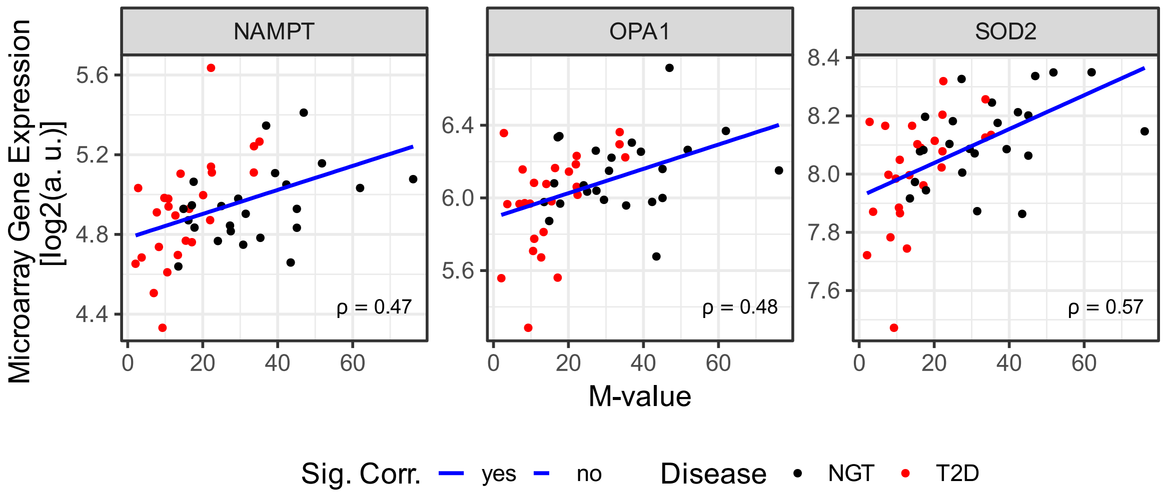

Figure S6. Spearman correlation (ρ) analysis between M-values and the microarray basal gene expression for *NAMPT, OPA1* and *SOD2*. All Spearman correlations were significant (FDR < 0.05). Black dots: normal glucose tolerant (NGT), red dots: Type-2 diabetic (T2D).

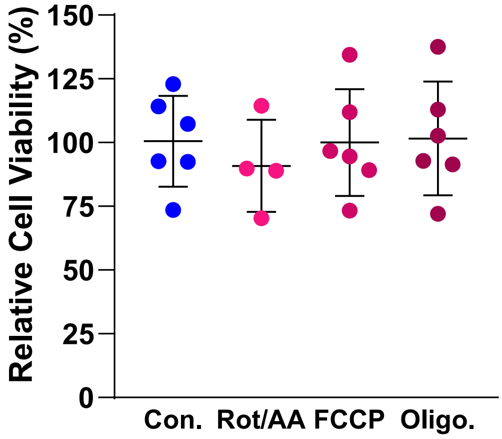

Figure S7. Lactate Dehydrogenase (LDH) was assessed in primary myocytes from NGT donors (n=4-6 donors). Cells were treated with either vehicle control (Con.) Rotenone/Antimycin A [(0.38 µm) Rot/AA], FCCP (2 µm) and Oligomycin [(1 µm) Oligo]. Values are shown as a percentage relative to Con. One-way ANOVA detected no differences between conditions.

table s4. Clinical Characteristics of the Donors for Muscle Cell Culture Studies.

	NGT		T2D		
	**Mean**	**SEM**	**Mean**	**SEM**	**p-value**
**Age**	62.6	1.1	60	1.4	**0.005**
**Weight [kg]**	93.7	3.6	89.7	6.3	0.174
**Height [cm]**	180.3	1.9	180.0	3.6	0.875
**BMI [kg/m^2]**	28.8	0.8	27.6	1.2	**0.012**
**BP Systolic [mm Hg]**	139.6	3.3	138.2	8.9	0.716
**BP Diastolic [mm Hg]**	87.9	3.0	81.5	5.4	**0.022**
**Pulse [BPM]**	61.5	4.5	65.3	4.1	0.099
**HbA1c [mmol/mol]**	30.3	1.5	59.6	5.6	**<0.0001**
**Plasma-glucose 0 OGTT [mmol/l]**	5.14	0.20	9.06	0.85	**<0.0001**
**Plasma-glucose 120 OGTT [mmol/l]**	5.96	0.67	16.16	1.39	**<0.0001**
**Plasma-TG [mmol/l]**	1.41	0.38	1.25	0.26	0.084
**Plasma-Chol [mmol/l]**	5.83	0.37	3.84	0.36	**<0.0001**
**Plasma-HDL [mmol/l]**	1.37	0.16	1.22	0.08	**<0.0001**
**Plasma-LDL [mmol/l]**	3.83	0.35	2.00	0.25	**<0.0001**
**VO_2max [ml/min]**	2478	207	2735	361	0.194
**VO_2max [ml/kg/min]**	28.7	2.0	31.6	2.6	0.153
**Max workload [Watt]**	181.9	16.8	193.8	26.3	0.454

Skeletal muscle biopsies were obtained from Normal Glucose Tolerant (NGT; n=7) and Type 2 Diabetic (T2D; n=5) men and myotube cultures were derived for *in vitro* studies. Results are mean±SEM. P-values were determined by Student's *t*-test.

table S5. Clinical Characteristics of the Participants in the Hyperinsulinemic-Euglycemic Clamp Study.

	NGT		T2D		
	**Mean**	**SEM**	**Mean**	**SEM**	**p-Value**
**Age**	57.2	2.3	61.8	1.5	0.093
**Weight [kg]**	83.6	2.3	88.0	1.7	0.137
**Height [m]**	1.8	0.0	1.8	0.0	0.127
**BMI [kg/m^2]**	26.7	0.4	27.3	0.5	0.373
**Waist [cm]**	96.1	1.9	98.7	1.3	0.270
**Hip [cm]**	100.3	1.2	102.0	1.1	0.290
**W/H ratio**	0.96	0.0	1.0	0.0	0.397
**Body fat [%]**	22.9	1.1	24.0	1.0	0.437
**BP Systolic [mm Hg]**	137.7	2.4	143.6	2.8	0.112
**BP Diastolic [mm Hg]**	83.3	1.4	83.9	1.7	0.793
**Pulse [BPM]**	63.2	2.2	67.3	1.5	0.132
**Fasting plasma-Glucose [mmol/l]**	5.6	0.2	7.8	0.3	**0.000**
**HbA1c [mmol/mol]**	36.5	0.7	50.9	1.2	**0.000**
**Fasting serum-Insulin [pmol/l]**	49.4	6.9	71.3	7.7	**0.039**
**HOMA1-IR**	1.8	0.3	3.6	0.4	**0.001**
**M-Value [µmol/(kg*min)]**	34.0	3.2	15.3	1.9	**0.000**
**Creatinine [µmol/l]**	85.6	1.9	82.6	3.2	0.435
**ASAT [µkat/l]**	0.4	0.0	0.4	0.0	0.568
**ALAT [µkat/l]**	0.4	0.0	0.5	0.0	0.502
**Plasma TG [mmol/l]**	1.2	0.2	1.2	0.2	0.731
**Plasma Chol [mmol/l]**	5.3	0.2	4.5	0.2	**0.003**
**HDL Chol [mmol/l]**	1.4	0.1	1.3	0.1	0.116
**LDL Chol [mmol/l]**	3.7	0.3	2.6	0.2	**0.001**
**FFA [mmol/l]**	0.5	0.0	0.5	0.0	0.585
**C-Peptide [nmol/l]**	0.7	0.1	0.9	0.1	**0.008**

The study groups include men with normal glucose tolerance (NGT; n=24), as determined by oral glucose tolerance test, or T2D diagnosis (n=25). Skeletal muscle biopsies were obtained for gene expression analysis. Insulin sensitivity (M-value) was determined by the hyperinsulinemic-euglycemic clamp. Results are mean±SEM. P-values were determined by Student's *t*-test.

table S6. Primer Sequences and Assay IDs used for the Gene Expression Analysis.

**SYBR™ Human**		
**Gene**	**Sequence**	
ARNTL	**Fwd**	ACCACAAGAACTTCTAGGCAC
	**Rev**	CCAGGACGTTGGCTAAAACA
NPAS2	**Fwd**	TGAATCTGACCACACCTGCT
	**Rev**	CTCTGGGCGTACTTGACTTG
NR1D1	**Fwd**	CTGGGAGGATTTCTCCATGA
	**Rev**	TCACTGTCTGGTCCTTCACG
PER2	**Fwd**	CGTGTCAGTGTCCGGAAAAG
	**Rev**	GGAGGAATTCTAGGGGCTTCA
B2M	**Fwd**	ATATAAGTGGAGGCGTCGCG
	**Rev**	TGAATCTTTGGAGTACGCTGG
GUSB	**Fwd**	GCAGATGTGTGACCGCTATG
	**Rev**	TGAGCGATCACCATCTTCAAG
TBP	**Fwd**	AGTTCTGGGATTGTACCGCA
	**Rev**	TATATTCGGCGTTTCGGGCA
RPLO	**Fwd**	TGGAGAAACTGCTGCCTCAT
	**Rev**	GATTTCAATGGTGCCCCTGG

**TaqMan™**	
**Gene**	**Assay ID**
	**Mouse**
B2m	Mm00437762_m1
Gapdh	Mm99999915_g1
Opa1	Mm01349707_g1
Arntl	Mm00500226_m1
Clock	Mm00455950_m1
Nr1d1	Mm00520708_m1
Per2	Mm00478099_m1
Per3	Mm00478120_m1
	**Human**
OPA1	Hs01047018_m1
TBP	Hs00427620_m1
B2M	Hs00187842_m1
PER3	Hs00213466_m1
